# Regulation of *Melanophilin* (*Mlph*) gene expression by the glucocorticoid receptor (GR)

**DOI:** 10.1038/s41598-021-96276-w

**Published:** 2021-08-19

**Authors:** Cheol Hwan Myung, Ji Eun Lee, Chan Song Jo, Jong il Park, Jae Sung Hwang

**Affiliations:** Department of Genetic Engineering & Graduate School of Biotechnology, College of Life Sciences, Kyung Hee University, Gyeonggi-do, 17104 Republic of Korea

**Keywords:** Cell biology, Molecular biology

## Abstract

Mlph plays a crucial role in regulating skin pigmentation through the melanosome transport process. Although Mlph is a major component involved in melanosome transport, the mechanism that regulates the expression of the *Mlph* gene has not been identified. In this study, we demonstrate that Mlph expression is regulated by the glucocorticoid receptor (GR). Alteration of GR activity using a specific GR agonist or antagonist only regulated the expression of Mlph among the 3 key melanosome transport proteins. Translocation of GR from the cytosol into the nucleus following Dex treatment was confirmed by separating the cytosol and nuclear fractions and by immunofluorescence staining. In ChIP assays, Dex induced GR binding to the Mlph promoter and we determined that Dex induced the GR binding motif on the Mlph promoter. Our findings contribute to understanding the regulation of Mlph expression and to the novel role of GR in *Mlph* gene expression.

## Introduction

Melanosomes are unique organelles where melanin pigments are synthesized and stored by maturation during transport^1^. Melanosome transport involves the intracellular distribution of melanosomes and their movement from around the nucleus to the dendrite tips of melanocytes^2, 3^. Rab27a, Mlph and MyoVa form a tripartite complex that is well known to be critically involved in melanosome transport through actin filaments^4–8^. When any one of those melanosome transport proteins is impaired, melanosome distribution is disrupted, which results in melanosome aggregation around the nucleus^9–11^.

A previous study reported that Mlph is induced by the mineralocorticoid receptor (MR) in murine cortical collecting duct cells after aldosterone treatment^43^. That study suspected that the increased level of Mlph (by aldosterone) may increase epithelial Na + channel (ENaC) trafficking and promote melanosome transport, although that group used different cells (cortical collecting duct cells rather than melanocytes) and did not confirm melanosome distribution or the expression of other melanosome transport proteins. The structure of the GR agonist is similar to aldosterone so it also makes sense that a GR agonist also regulates Mlph expression.

GR is a well-known member of the nuclear receptor family. GR consists of 777 amino acids and serves as a transcription factor or enhancer that affects the expression of numerous target genes^12^. GR is typically localized in the cytosol in an inactive state due to its combination with heat-shock proteins (HSPs) and various factors^13^. Specific GR agonists, such as Dex or cortisol, bind to the ligand-binding domain (LBD) of GR and dissociate it from the HSP complex. Ligand bound GR has an exposed internal nuclear localization signal (NLS), which leads to the translocation of GR into the nucleus^14, 15^.

Various factors in fetal bovine serum (FBS) have been added to culture medium to facilitate cell proliferation and to supply nutrition^16, 17^. FBS contains cortisol, a GR agonist, which originates from a series of signal transductions from the Hypothalamic–pituitary–adrenal (HPA) axis. Cortisol is secreted into blood vessels^18^. In this study, we used serum-free medium or charcoal-stripped serum in the culture medium to prevent the effects of undefined levels of cortisol in the serum. The exception to this was the use of FBS during subculture of melanocytes to maintain their high growth rates. We also confirmed how cortisol in the serum affects MIph expression and how melanosome transport is exhibited.

Previous studies of melanosome transport proteins have focused on their interactions^19–26^. The mechanisms of regulating the expression of their encoding genes are not well understood. Several substances affecting the interactions of transport proteins have been studied in efforts to control melanosome distribution^27, 28^. A recent study found that the transcription factor MITF, the master melanocyte regulator, activates Rab27a gene expression and melanin synthesis^29^. However, the mechanism that regulates *Mlph* gene expression in melanocytes is not well understood.

We previously reported that 2-methylnaphtho[1,2,3-de]quinolin-8-one (MNQO) and 16-kauren-2beta-18,19-triol ^16-kauren^ inhibit the expression of melanosome transport proteins, ultimately inhibiting skin pigmentation in human artificial skin and guinea pig skin models^30, 31^ MNQO repressed the expression of Rab27a, Mlph and MyoVa while 16-kauren specifically reduced Mlph expression, which led to the inhibition of pigmentation.

In this study, we investigated the specific transcription factor that regulates the *Mlph* gene and the specific regulatory mechanism of Mlph expression among melanosome transport proteins. GR agonist induces GR binding to IR9(− 213/− 192) of Mlph promoter and increases Mlph expression. The results demonstrate that Mlph is regulated by GR activity, and that GR serves as a transcription factor of the *Mlph* gene.

## Results

### The Dex induced activation of GR promotes melanosome distribution

**Figure 1:**
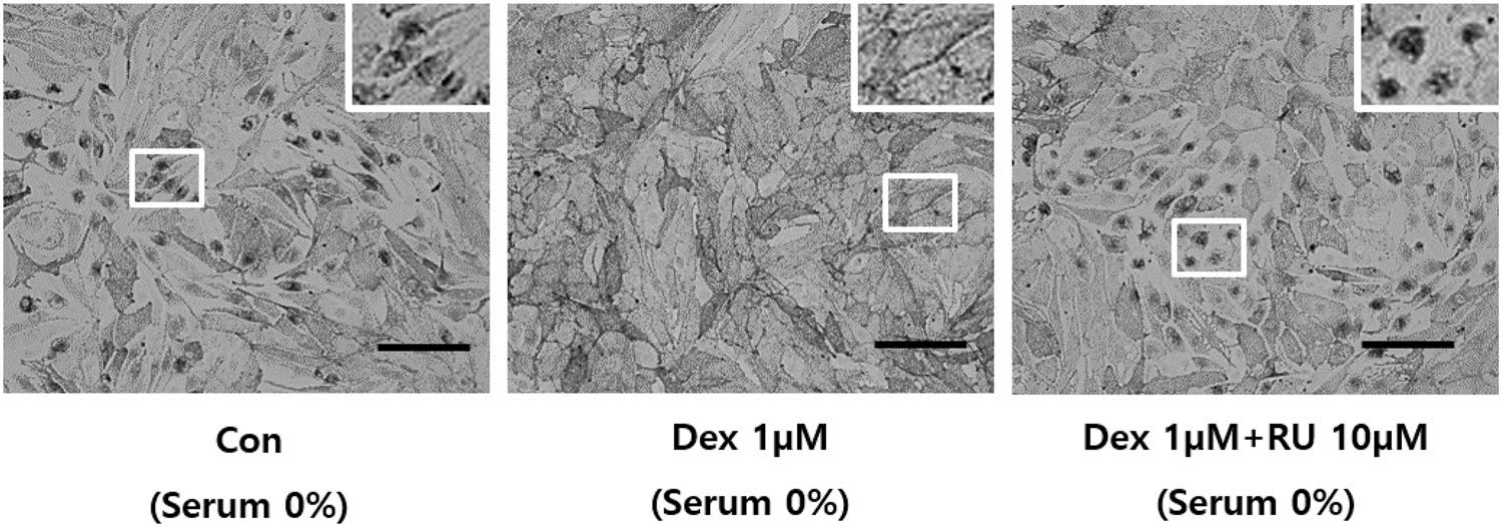
The GR agonist, Dex, induces melanosome movement. Melan-a melanocytes were cultured in serum-free medium with or without pretreatment for 30 min with 10 µM Ru486 before treatment with or without 1 µM Dex for 72 h. Melanosome distribution was observed using bright field microscopy at × 400 magnification. Higher magnification images of the areas denoted by white boxes are located at the upper-right of each panel. (scale bar = 100 µm).

To confirm whether GR is involved in regulating melanosome transport, we validated the melanosome distribution in Melan-a melanocytes cultured in serum-free medium using Dex and RU486, a specific GR agonist and antagonist, respectively. Most melanosome aggregation disappeared when melanocytes were cultured with Dex, and the melanosomes of Dex-treated Melan-a melanocytes were distributed in the whole cell body compared with melanocytes pre-exposed to RU486 or cultured in serum-free medium (Fig. 1). As a result, melanosomes without GR activation were aggregated around the nucleus, while GR activation by Dex induced melanosome transport. This result indicates that melanosome transport in melanocytes is regulated by GR protein activity.

### GR activation induces melanosome transport by upregulating Mlph

**Figure 2:**
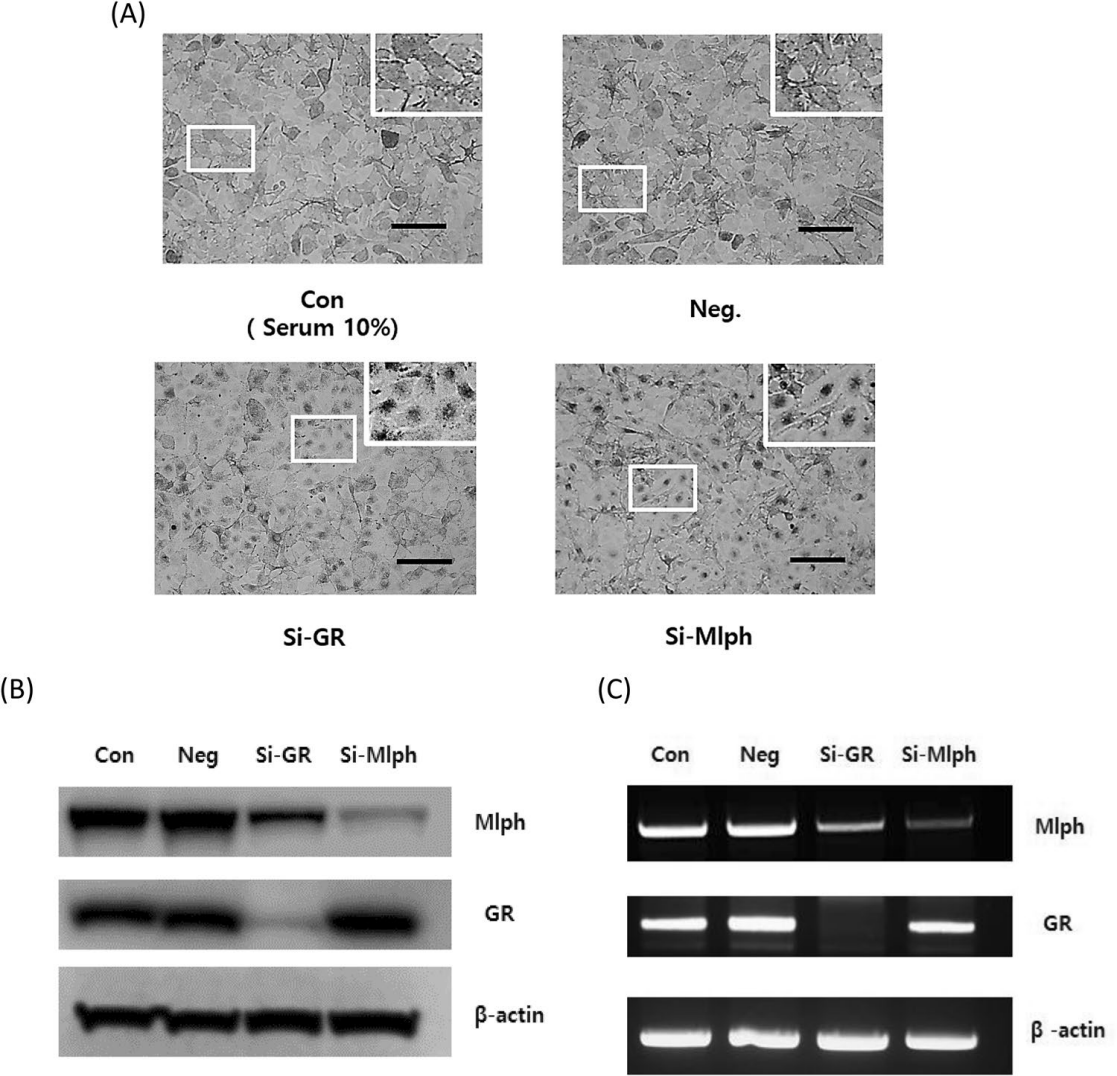
Knockdown of GR inhibits melanosome distribution. (**A**) Melanosome distribution in Melan-a melanocytes after knockdown of GR. Melan-a melanocytes were transfected with negative control, GR or Mlph siRNA for 24 h, then were replenished with 10% FBS medium and cultured further for 72 h. Melanosome transport was observed using bright field microscopy. Higher magnification images of the areas denoted by white boxes are located at the upper-right of each panel. (**B**) Protein expression levels of Mlph and GR after knockdown of GR. Melan-a melanocytes were replenished with 10% FBS medium and cultured for 72 h after each siRNA transfection. Proteins were measured by western blotting. (**C**) mRNA expression levels of Mlph and GR. After siRNA transfection, Melan-a melanocytes were cultured in 10% FBS medium for 24 h, after which mRNA expression levels were analyzed using RT PCR. (scale bar = 100 µm). All data were mean ± SD and from three independent experiments. Data were analyzed by Student’s t-test. **P < 0.01, ***P < 0.001. The band intensity was quantified using NIH ImageJ software 1.45 s and normalized relative to β-actin. Original images and quantification were provided in Supplemental file.

To confirm that GR plays a crucial role in regulating melanosome transport, we used a siRNA to knockdown GR, and then evaluated the effects on melanosome movement. Knockdown of GR in melanocytes resulted in melanosome aggregation as did a Mlph siRNA used as a positive control (Fig. 2A). The effect of GR on MIph protein expression was assessed by western blotting, which revealed that the inhibition of melanosome transport was attributable to the downregulation of Mlph protein (Fig. 2B). Similarly, at the transcriptional level, the knockdown of GR reduced the expression of MIph mRNA as measured by RT-PCR (Fig. 2C). Those findings indicate that GR regulates the expression of Mlph at the transcriptional level, and suggest that it acts as a transcription factor for the *Mlph* gene.

**Figure 3:**
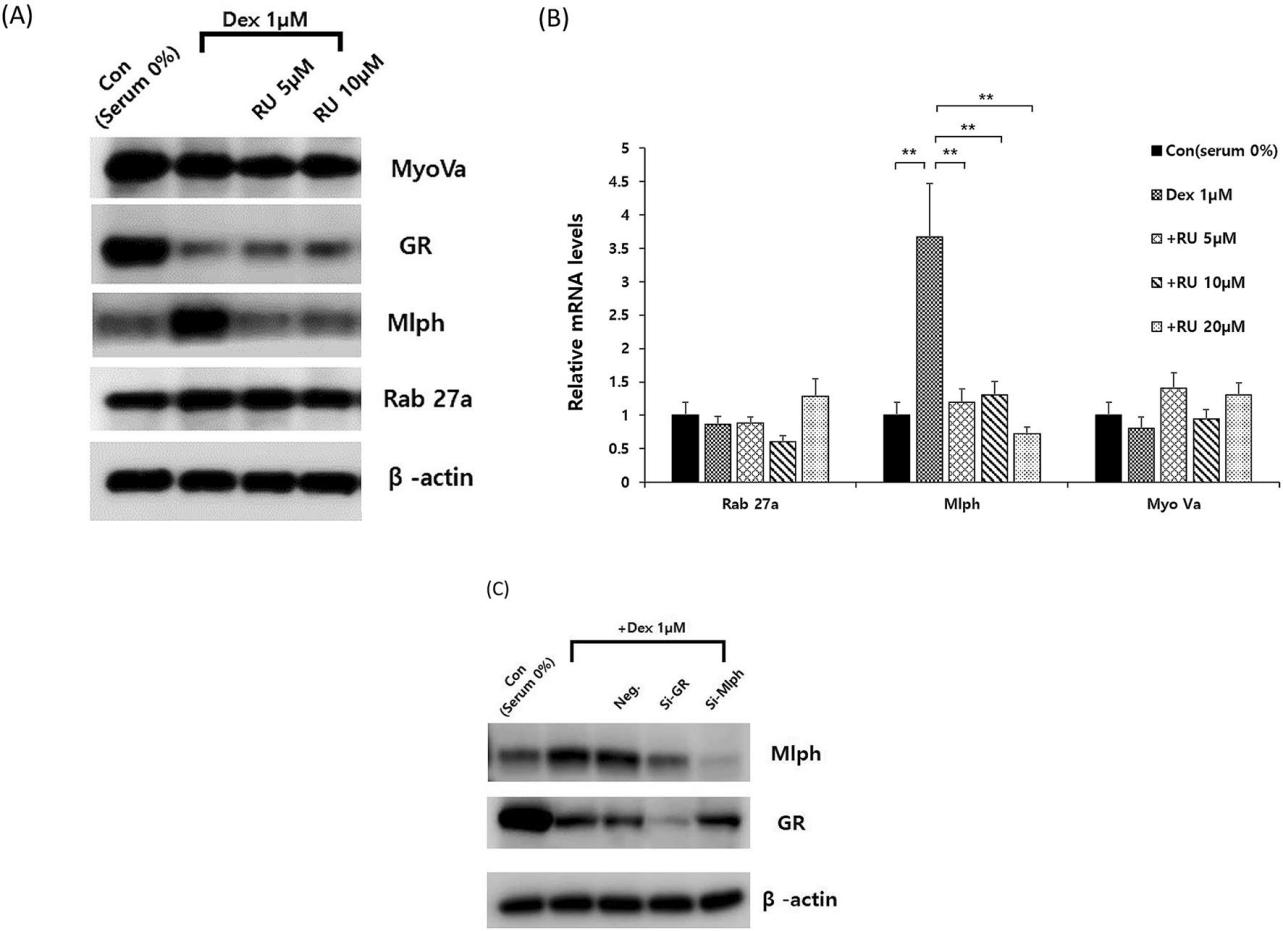
GR activation only regulates Mlph expression among the melanosome transport proteins. (**A**) Expression levels of melanosome transport proteins. Melan-a melanocytes were cultured in serum-free media with 1 µM Dex for 72 h or with RU486 at the concentration noted for 30 min before Dex exposure, after which cell extracts were subjected to western blotting. (**B**) mRNA expression levels of melanosome transport proteins. Melan-a melanocytes were cultured in serum-free medium with 1 µM Dex for 24 h with or without RU486 pretreatment for 30 min. mRNA expression levels were quantified by q-PCR. (**C**) Effect of altering GR activity on GR and Mlph expression. Melan-a melanocytes were cultured in serum-free medium with 1 µM Dex for 72 h after GR and Mlph siRNA transfection. Protein expression was measured using western blotting. All data in this figure represent the mean ± SD of three independent experiments. Data were analyzed by Student’s t-test. **P < 0.01, ***P < 0.001. The band intensity was quantified using NIH ImageJ software 1.45 s and normalized relative to β-actin. Original images and quantification were provided in Supplemental file.

The melanosome transport process involves Rab27a, MyoVa and Mlph, which form a tripartite complex that moves along actin filaments. The expression of Rab27a, MyoVa and Mlph in Melan-a melanocytes was assessed following exposure to a GR agonist or antagonist. This experiment sought to determine whether GR specifically regulates Mlph expression without affecting the expression of other melanosome transport proteins. Melan-a melanocytes were pretreated with or without RU486 before treatment with or without Dex, and the subsequent mRNA expression was analyzed by q-PCR and protein expression was analyzed by western blot (Fig. 3A,B). Only the mRNA and protein expression levels of Mlph among all 3 transport proteins responded to changes in GR activity elicited by Dex or RU486. In contrast, the expression of Rab27a and MyoVa was independent of GR activity. This result means that GR is a specific regulator of Mlph, and is not other members of the tripartite complex.

Dex and RU486 downregulated GR expression in melanocytes (as demonstrated by western blotting) (Fig. 3A). It had been previously described that variations in GR activity reduce GR expression through autoregulation^33, 34^. We evaluated the activity of GR through Mlph expression following Dex treatment and GR knockdown using siRNA. MIph protein expression was significantly increased by Dex despite the downregulation of GR via autoregulation of the GR agonist (Fig. 3C). Conversely, treatment with Dex failed to increase Mlph expression when GR knockdown occurred. This latter result suggests that GR is in an active state and simultaneously increases Mlph expression even though treatment with Dex decreases GR through autoregulation.

### Serum cortisol induces melanosome transport via the upregulation of MIph

There are many undefined factors in FBS that facilitate cell proliferation in culture medium. Cortisol in the FBS is a GR agonist that is secreted from the adrenal cortex and is circulated in blood vessels in vivo^35^. We evaluated the effect of cortisol on Mlph expression. When FBS was treated with dextran coated charcoal to remove endogenous steroid hormones such as cortisol, melanosome distribution was predominantly inhibited (Supplemental Figure S1). In addition, treatment with 10% FBS or Dex promoted melanosome transport compared to the serum-free medium. In contrast, melanosomes were aggregated in the presence of RU486. Melanosome transport was inhibited by reduced Mlph protein expression (Supplemental Figure S2). Therefore, GR activation by cortisol in the FBS plays an important role in Mlph expression, and subsequently in melanosome transport. Contrary to our expectations, melanosome transport was not inhibited when melanocytes were cultured in the charcoal stripped-FBS (Supplemental Figure S1). We found that removing endogenous steroids with the charcoal stripping process was not sufficient to suppress Mlph expression (Supplemental Figure S2). This finding suggests that the small amount of cortisol remaining in charcoal-stripped FBS is sufficient to activate Mlph expression.

### Dex promotes the translocation of GR into the nucleus

**Figure 4:**
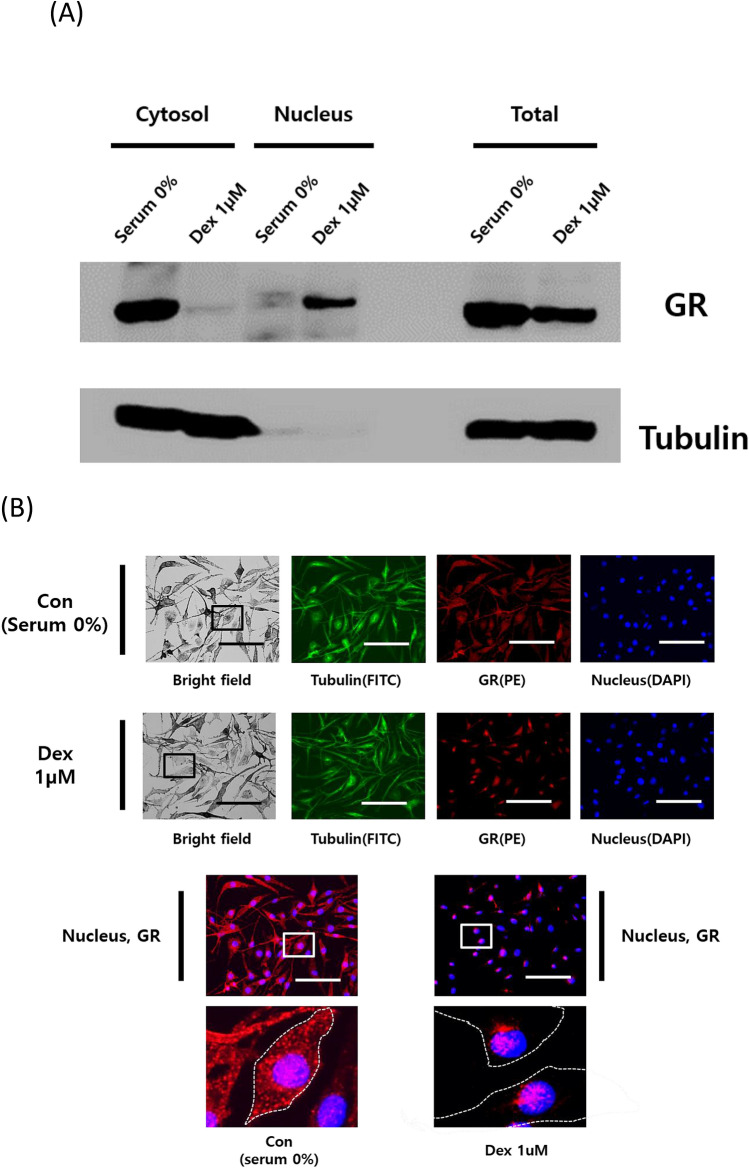
Dex induces GR translocation from the cytosol into the nucleus. (**A**) The effect of Dex on GR location. Melan-a melanocytes were cultured in serum-free media or with 1 µM Dex for 6 h, and cytosol and nuclear fractions were isolated. Identification of GR localization was measured by immunoblotting. (**B**) Effect of Dex on GR localization. Melan-a melanocytes were cultured in serum-free medium or treated with Dex were subjected to immunofluorescence analysis. Melanosome distribution was identified using bright field microscopy. Tubulin, GR and nuclei were identified by FITC, PE and DAPI, respectively. Merged images show the co-localization of GR and nuclei. White dotted lines indicate cell shapes. (scale bar = 100 µm). All data were mean ± SD and from three independent experiments. Data were analyzed by Student’s t-test. **P < 0.01, ***P < 0.001. The band intensity was quantified using NIH ImageJ software 1.45 s and normalized. Original images and quantification were provided in Supplemental file.

GR is a well-known transcription factor that is localized in the cytosol under normal conditions, but it translocases to the nucleus to regulate its target genes in the presence of a GR agonist^36^. We isolated cytosol and nuclear fractions of melanocytes to determine if GR is translocated from the cytosol to the nucleus by treatment with Dex. Dex treatment led to the translocation of GR from the cytosol into the nucleus (Fig. 4A). In accordance with our earlier results, autoregulation reduced the amount of total GR expression. We then performed immunofluorescence microscopy to confirm this result visually (Fig. 4B). Melanosome distribution (Black) was distinguished in bright field microscopy, while cytoskeletal tubulin and GR were distinguished using immunofluorescence staining with FITC (Green) and PE (Red), respectively. Treatment with Dex induced GR accumulation in the nuclei. In contrast, in the control, GR was localized throughout the cell body including the nucleus. GR that translocated into the nucleus can act as a transcription factor that regulates transcription via GR binding sites or GR tethering sites.

### GR directly regulates *Mlph* gene expression

**Figure 5:**
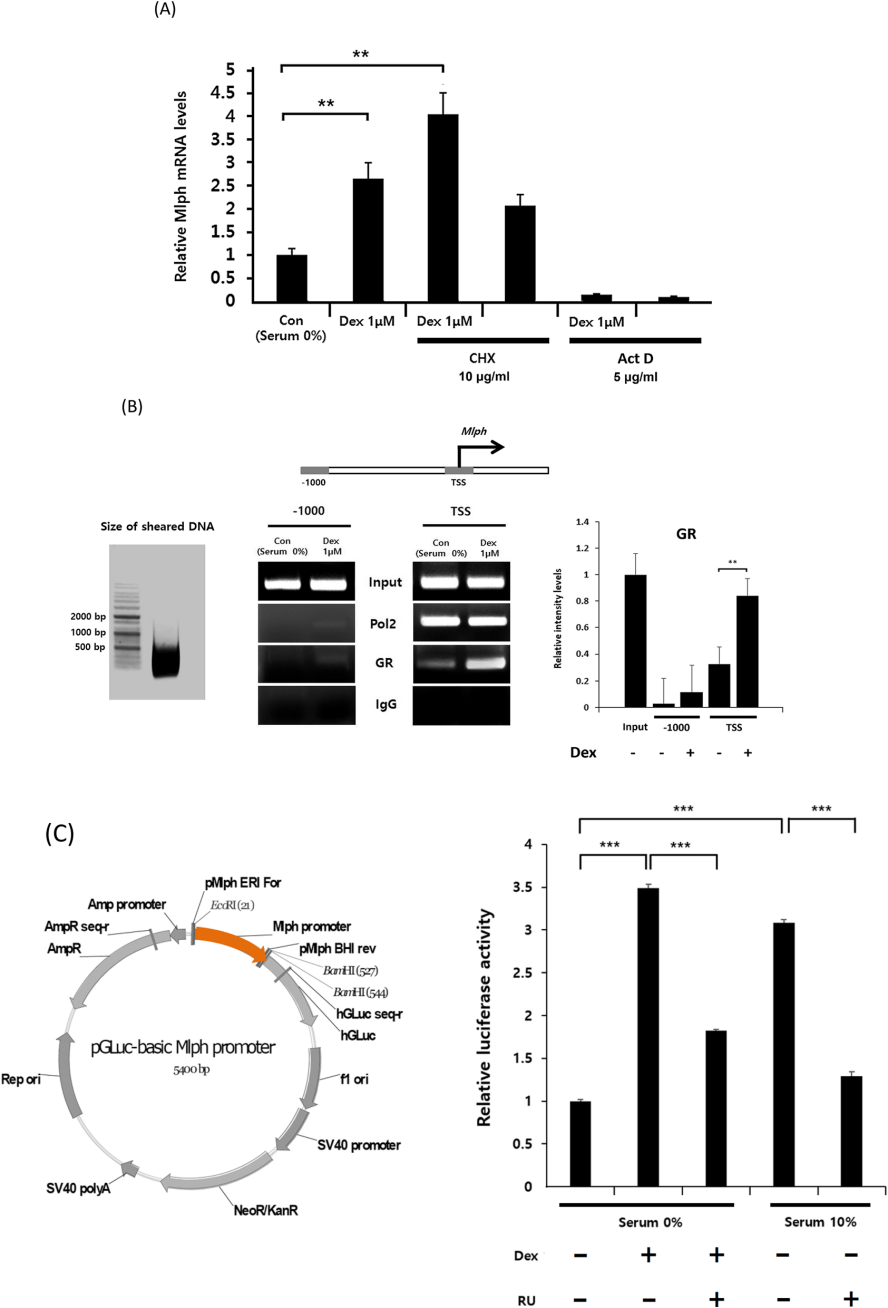
GR regulates Mlph expression via direct promoter binding. (**A**) GR activation is involved in Mlph expression without protein synthesis. Melan-a melanocytes were cultured in serum-free medium or were pretreated with CHX 10 μg/ml or Act D 5 μg/ml for 2 h, then replenished with serum-free media including the indicated concentration of Dex for 24 h. Mlph mRNA was quantified by q-PCR. (**B**) Binding affinity of GR to the Mlph promoter. Melan-a melanocytes were cultured in serum-free medium with or without 1 µM Dex for 24 h. Chromatin extracts were sheared into < 500 bp. Agarose beads separated fragments bound with Pol2, GR and IgG from extracts. GR binding affinity was quantified by PCR using primers of TSS and -1000 region of the Mlph promoter. (**C**) The effect of GR activity on luciferase activity containing the Mlph promoter. The luciferase vector includes 517 bp of the Mlph promoter and was transfected into Melan-a melanocytes. Using selection with G418, a stably luciferase expressing cell line was obtained. Luciferase activity was measured following Dex treatment for 72 h with or without pretreatment with RU486. All data in this figure represent the mean ± SD of three independent experiments. Data were analyzed by Student’s t-test. **P < 0.01, ***P < 0.001. The band intensity was quantified using NIH ImageJ software 1.45 s and normalized. Original images were provided in Supplemental file.

We used cycloheximide (CHX), a translation inhibitor^37^, to determine whether GR regulates Mlph expression directly or through de novo factors or signals. Actinomycin D (Act D)^38^, a transcription inhibitor used as a positive control, significantly inhibited the transcription of Mlph (Fig. 5A). In the presence of CHX, Mlph mRNA levels were still increased by Dex without de novo protein synthesis. This result means that GR directly induces Mlph expression at the transcriptional level without any effect on other protein synthesis. The mRNA expression level of Mlph increased approximately twofold following treatment with CHX alone. This phenomenon suggests that CHX represses the expression of proteins related to Mlph mRNA degradation (either directly or indirectly through signals associated with Mlph mRNA degradation)^34^.

In order to confirm whether GR can directly bind to the Mlph promoter, we examined the binding affinity of GR to the transcription start site (TSS) and the − 1000 region of the Mlph promoter. Melan-a melanocytes treated with or without Dex were crosslinked by formaldehyde, after which the crosslinked DNA was sheared into small fragments < 500 bp using a sonicator (Fig. 5B, left). The results show that GR, as well as RNA polymerase2 (pol2), directly bound to the TSS of the Mlph promoter. Treatment with Dex stimulated the binding affinity of GR to the TSS (Fig. 5B). In contrast, binding of GR or pol2 to the − 1000 region was weak. The small amount of pol2 and GR binding had a tendency to increase with Dex treatment. These results suggest that GR binds to the TSS of Mlph within 500 bp.

We previously reported that 16-kauren, a specific Mlph inhibitor, reduced luciferase expression containing 517 bp of the Mlph TSS^31^. Accordingly, Melan-a melanocytes were used to determine whether GR affects the Mlph promoter within 517 bp. In serum-free medium, luciferase activity was increased by 3.5-fold following Dex treatment but in contrast, the luciferase activity was not increased by pretreatment with RU486 (Fig. 5C). Likewise, luciferase activity was higher in the FBS medium cultured melan-a cells containing an undefined level of cortisol than it was in serum-free medium and not increased by pretreatment with RU486. These data show that GR can regulate Mlph expression via the 517 bp Mlph promoter.

### GR is capable of binding to a specific sequence in the Mlph promoter

**Figure 6:**
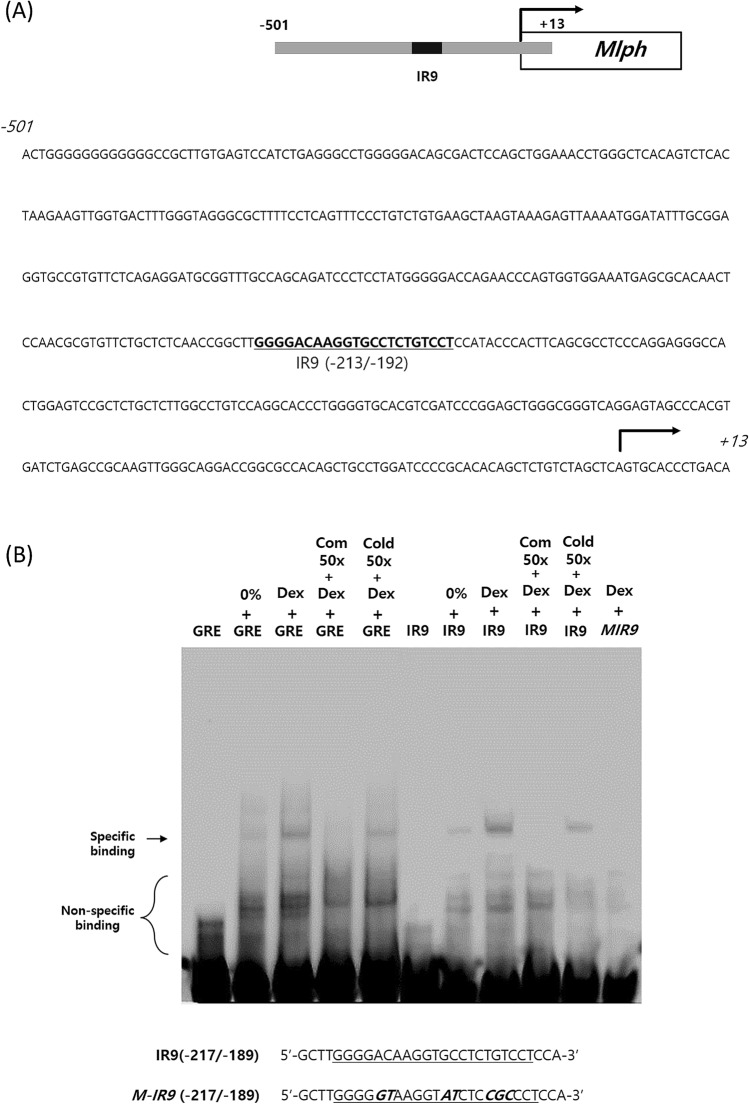
Dex increases GR binding to IR9 of the Mlph promoter. (**A**) DNA sequence of the Mlph promoter. Sequence shows the Mlph promoter from TSS to -501. IR9 represents one of the high ranked GR binding sites predicted by NUBIscan. (**B**) Effect of Dex induced-alteration of GR activity on binding affinity to IR9 of the Mlph promoter. Melan-a melanocytes were cultured in serum-free medium with or without 1 µM Dex. Cell nuclear extracts were reacted with biotin-labelled GRE, IR9 or mutated IR9 (M-IR9); a 50-fold excess of each unlabeled probe was used as a competitor. Binding reactions proceeded in ice for 1 h and binding of GR and the probe was confirmed by electrophoretic band shift. All data in this figure represent the mean ± SD of three independent experiments. Data were analyzed by Student’s t-test. **P < 0.01, ***P < 0.001. The band intensity was quantified using NIH ImageJ software 1.45 s and normalized. Quantification of band-shift was provided in Supplemental file.

We attempted to identify the GR binding sequence of Mlph and we searched for a GR binding site within approximately 500 bp upstream of the Mlph TSS. This area was searched based on the ChIP assay and luciferase assay results, which showed that GR was involved in Mlph expression within 500 bp from the TSS (Fig. 6A). We identified numerous GR occupied regions within 517 bp of the Mlph promoter using the ‘NUBIscan (https://www.nubiscan.unibas.ch/)’ program (Supplemental Figure S3B).

Therefore, we sought to determine the sequence(s) where binding affinity was augmented by activated GR in the presence of Dex. In the Dex-treated cell nuclear extract, the GR binding affinity to the 3^rd^ part (− 229/− 189) was increased (Supplemental Figure S4 and S5). We also used NUBIScan which is the searching program of transcription factor binding site, and found the 45 binding candidate sites (supplemental Table 1). The 3rd part (− 229/− 189) coincided with IR9 (Inverted Repeat, − 213/− 192, 21 bp), which is a highly ranked site by the NUBIScan program (Fig. 6A). We confirmed whether the GR binding to IR9 is sequence specific. We used IR9 (− 217/− 189, 29 bp including − 213/− 192, 21 bp) due to the comparison GR binding band shift with a similar size of GRE (33 bp, positive). Some of free GR that doesn’t bind to proteins such as heat shock proteins can also enter the nucleus cultured in serum free medium (Fig. 6B, lane 2, 7). Dex promoted binding between the GR of the nuclear extract and biotin-labelled IR9. The results also showed that a 50-fold excess of biotin unlabeled probe as a competitor interfered with the biotin labelled GR binding band. In contrast, a 50-fold excess of the cold sequence did not. In addition, a 7 bp mutated IR9 was not capable of binding GR. Therefore, we determined that Dex increases the binding of nuclear GR with (− 213/− 192) the Mlph promoter.

**Figure 7:**
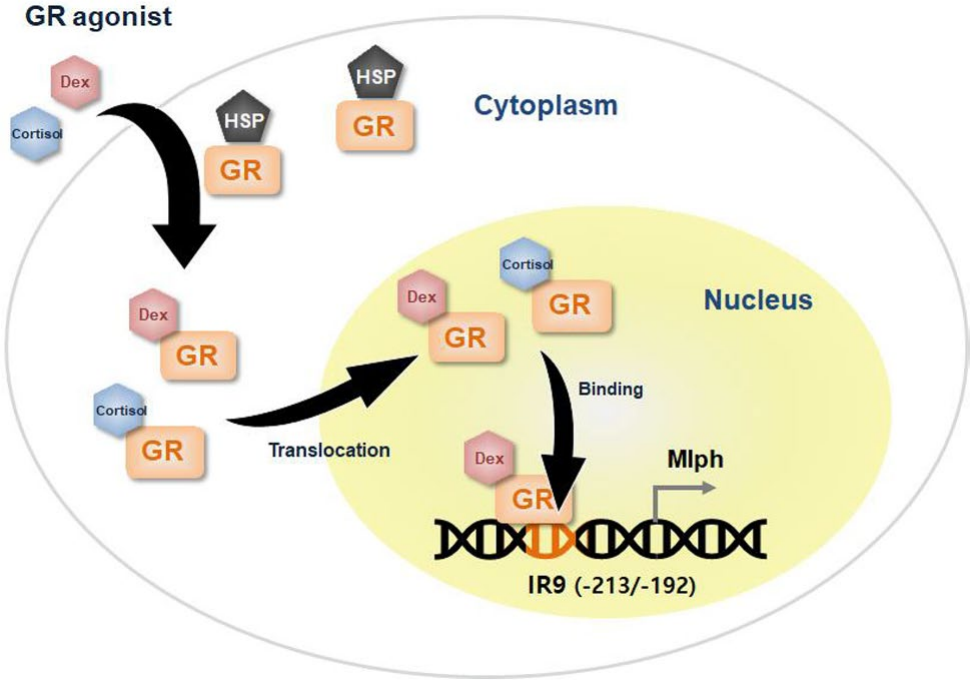
Scheme of GR regulation on *Mlph* gene expression.

Our results suggest that GR is a transcription factor that directly binds to the Mlph promoter. GR is typically in an inactive state and is localized in the cytosol in a HSP complex. Treatment with Dex dissociates GR from the HSP complex and allows it to translocate to the nucleus. There are numerous GR binding candidates within 500 bp of the Mlph promoter. However, Dex induces GR binding affinity to IR9 (− 213/− 192) of the Mlph promoter (shown schematically in Fig. 7).

## Discussion

Dysfunctions of any protein involved in melanosome transport prevent melanosome distribution and pigmentation (data not shown), because melanosome transport can only occur when Rab27a, Mlph and MyoVa form a functional tripartite complex^7^. Mlph plays a critical role in that complex as a linker between Rab27a and MyoVa. Therefore, it is very important to identify the mechanism that regulates *Mlph* gene expression in order to understand the complex processes involved in modulating skin pigmentation. However, Mlph expression and its regulation are still poorly understood. Our results show that GR regulates Mlph expression. In addition, a GR agonist increases the binding affinity of GR to the IR9 sequence of the Mlph promoter.

Mlph mRNA levels were increased by twofold by treatment with Dex compared to the control despite treatment with CHX alone, which is a translation inhibitor (Fig. 5A). This phenomenon may be due to the inhibited expression of a Mlph transcription repressor and/or mRNA degradation-related proteins. Interestingly, the increased Mlph mRNA level elicited by CHX was also found following treatment with Dex. This finding indicates that GR activation is not related to the expression of a Mlph repressor or a mRNA degradation-related protein, and GR directly regulates the expression of Mlph.

ChIP assays revealed that GR binds weakly to the − 1000 region of Mlph, as well as the TSS (Fig. 5B). These findings suggest that there are other factors interacting with IR9 bound GR. Indeed, the GR protein is well known for tethering with other transcription factors, as well as serving as a transcription factor that binds directly to the promoter^15^. Therefore, it seems possible that the existence of GR, which interacts with the − 1000 region, is weakly or tethered to other transcription factors. We hypothesized that if another transcription factor is located at the − 1000 region, it would likely play the role of an Mlph repressor, because Mlph expression was increased by CHX treatment, which inhibits translation. However, further investigation is needed to define the interactions between GR and other factors in the Mlph promoter.

Initially, 517 bp of the Mlph promoter were selected as candidates for the GR binding region based on luciferase assays (which contained 517 bp of the Mlph promoter in our previous study)^23^. In addition, GR binds to the Mlph promoter that is sheared into a small size (< 500 bp) in ChIP assays, and Dex affects luciferase activity containing 517 bp of the Mlph promoter (Fig. 5B and C).

In preliminary experiment, there were at least 5 portions of GR binding candidates within 517 bp of the Mlph promoter in mobility shift assay (data not shown). This result coincides with the outcome of the NUBIscan program, which identified 45 potential binding sites (Supplemental Table S1). Therefore, we thought that the key point was to identify the sequence(s) that increased the binding affinity to GR in the presence of Dex. Dex-treated nuclear extracts increased binding with biotin labelled (− 297/− 189) (Supplemental Figure S4). As a result of more subdivision, we found that Dex promoted GR binding to the IR9 region, which was consistent with the highest ranked sequence (− 213/− 192, 21 bp) in the NUBIscan analysis (Supplemental Figure S3 and S5). Although we found that Dex treatment facilitated GR binding to specific sequences, further evaluations are needed to determine whether other GR binding candidates on the Mlph promoter are needed to affect Mlph expression. In addition, it is necessary to conduct an experiment using a luciferase reporter gene containing mutated IR9 to confirm IR9 sequence is the main element on Mlph expression.

GR agonists induce GR activation, but also downregulate GR expression through autoregulation^39^. However, we thought it was paradoxical that GR activates a target gene during the GR downregulation by Dex. Therefore, we performed GR knockdown to corroborate whether Dex induces GR activation despite the GR reduction. Dex did not increase Mlph expression following GR knockdown (Fig. 3C), meaning that GR is in a strongly active state despite its downregulation from autoregulation. Interestingly, changes in GR activity and expression elicited by Dex and RU486 did not affect the other melanosome transport proteins (Rab27a and MyoVa) (Fig. 3A,B). This result suggests that GR itself or GR-related sub signals are independent of Rab27a and MyoVa expression.

Reduction of GR (by its agonist) is a major problem causing GR resistance to therapeutics such as in rheumatoid arthritis^40–42^. In our study, GR protein expression levels were not decreased in medium containing 10% FBS even though its activity (with respect to the target gene) was comparable to cells cultured in serum-free medium with Dex (Supplemental Figure S2). This result reveals the possibility that the FBS contains not only cortisol, but also substances that strongly block GR autoregulation. Identification of these substances will be a breakthrough in the field of therapeutics for GR resistance diseases.

In this study, we demonstrated the regulatory mechanism of Mlph through direct GR binding to IR9 (− 213/− 192) of the Mlph promoter in the presence of Dex. Altogether, this study provides novel information regarding the regulatory mechanism of Mlph expression via GR.

## Materials and methods

### Materials

Dexamethasone (Dex, sigma, St. Louis, MO, USA), RU486 (RU, sigma), Cycloheximide (CHX, sigma), Actinomycin D (Act D, sigma).

### Cell culture

Murine Melan-a melanocytes were derived from C57BL/6 mice^32^ and were acquired from Dr. Dorothy Bennett (St. George’s Hospital, London, UK). Melan-a melanocytes were cultured in serum-free RPMI-1640 medium (Welgene, Kyungsan-si, Korea) supplemented with 1% penicillin/streptomycin (P/S) (Welgene) in all experiments. In order to maintain continuous growth, Melan-a melanocytes were cultured in RPMI-1640 medium supplemented with 10% FBS (Welgene), 1% P/S and 200 nM phorbol 12-myristate 13-acetate (PMA) (Sigma-Aldrich, St. Louis, MO, USA).

### Detection of melanosome aggregation

Melan-a melanocytes were seeded and cultured for 24 h for stabilization, then were pretreated where indicated in the text with RU486 10 µM (Sigma-Aldrich) before treatment with or without 1 µM Dex (Sigma-Aldrich) in RPMI-1640 containing 0% or 10% FBS and 1% P/S for 72 h. Each plate was observed using bright field microscopy. Images were photographed at × 200 magnification using a CELENA S Digital Imaging System (Logos Biosystems, Anyang-si, Korea). After 72 h of the indicated treatments, melanosome aggregation was evaluated by discriminating melanocytes with perinuclear melanosome aggregates into random microscopic fields per well.

### Western blotting

Melan-a melanocytes were lysed with RIPA buffer and 1X protease inhibitor cocktail (PIC) (Sigma-Aldrich). The protein extracts were separated at 200 V using SDS-PAGE and the separated proteins were then transferred to PVDF membranes (Pall, Glen Cove, NY, USA) at 25 V for 2 h. To analyze protein expression, the PVDF membranes were immunoblotted overnight at 4 °C with antibodies to β–actin (Sigma), MyoVa (Cell Signaling Technology, Danvers, MA, USA), Rab27a (Santa Cruz, Dallas, TX, USA), Mlph (Protein Tech Group Inc., Chicago, IL, USA), Tubulin (Cell Signaling Technology) and GR (Cell Signaling Technology). Secondary horseradish peroxidase (HRP)-conjugated anti-rabbit antibodies (Bethyl Laboratories, Montgomery, TX, USA) or anti-mouse antibodies (Bio-Rad, Hercules, CA, USA) were then incubated at room temperature for 1 h. Protein expression was detected using chemiluminescence ECL solution (Thermo, Waltham, MA, USA) and visualized with Chemi-Doc XRS (Bio-Rad).

### Small interfering RNA (siRNA)

siRNA oligonucleotides were synthesized by Bioneer (Daejeon-si, Korea). A negative siRNA, which includes sequences that are not related to the target gene, was purchased from Bioneer. The sense and antisense sequences for individual duplexes targeting mouse GR and Mlph were as follows:

GR siRNA sense 5ʹ-UGACUGCCUUACUAAGAAAUUd(T)d(T)-3ʹ and antisense 5ʹ-AAUUUCUUAGUAAGGCAGUCAd(T)d(T)-3ʹ and

Mlph siRNA sense 5ʹ-GGGCAAAAUACAAAAGGAGd(T)d(T)-3ʹ and antisense 5ʹ-CUCCUUUUGUAUUUUGCCCd(T)d(T)-3ʹ

### Reverse transcription (RT) PCR

Total RNA extracts of Melan-a melanocytes were isolated using TRIZOL (Takara, Shiga, Japan) according to the manufacturer’s protocol. The quality and quantity of each total RNA were measured using a NanoDrop2000 (Thermo Scientific). To obtain cDNA, 1 µg of each quantified total RNA was mixed with oligo(dT) (ELPIS, Daejeon-si, Korea), followed by denaturation at 65 °C for 5 min and chilling on ice for 5 min. The annealed samples were then mixed with reverse transcriptase and 2 mM dNTPs (Fermentas, Waltham, MA, USA) for 1 h at 42 °C. Reverse transcription was terminated at 70 °C for 10 min. For amplification, each cDNA was mixed with HiPi-PCR Mix (ELPIS). The primer sequences for the target genes were as follows:

GR sense 5ʹ-CTGGTGTGCTCCGATGAAGC-3ʹ and antisense 5ʹ-CTCCTTAATGTCACGCACGATTTC-3ʹ;

Mlph sense 5ʹ-ATGTAGACACCTCTGATGAAGA-3ʹ and antisense 5ʹ-TTAGGGCTGCTGGGCCATCAC-3ʹ

β-actin sense 5ʹ-GTGGGGCTGCCCCAGGCACCA-3ʹ and antisense 5ʹ-CCTAGAAGCATTTGCGGTGCACGATG-3ʹ

PCR products were analyzed by electrophoresis on 2% agarose gels in TAE buffer (ELPIS) and nucleic acids were stained with RedSafe (Intron, Sungnam-si, Korea).

### Quantitative real-time PCR

mRNA expression levels were quantified by quantitative real-time PCR using a Light Cycler (Roche, Mannheim, Germany) and a FastStart Essential Probes Master kit (Roche). The reactions were performed according to the manufacturer’s protocol. The following primers and probes were used:

Rab27a (#63, NM_023635.5), sense 5ʹ-GAAGACCAGAGGGCAGTGAA-3ʹ, antisense 5ʹ-ACTGGTTTCAAAATAGGGGATTC-3ʹ

Mlph (#108, NM_053015.3), sense 5ʹ-AGCCCCTCAACAGCAAAAA-3ʹ, antisense 5ʹ-TTCCTCAAAGTCCACATCTCʹ-3ʹ

MyoVa (#63, NM_010864.2), 5ʹ-GCGCCATCACCCTAAACA-3ʹ, and antisense 5ʹ-CCAGTTGACTGACATTGTACCTG-3ʹ

The primers and probes were designed using the Probe Library Assay (PLA) Design Center on the Roche homepage: ‘https://lifescience.roche.com/global_en/brands/universal-probe-library.html#assay-design-center’. All mRNA levels were normalized using β-actin mRNA levels.

### Luciferase assay

Melan-a melanocytes were transfected with a Gaussia luciferase reporter plasmid (pGLu-basic vector) containing the Mlph promoter region (− 500/ + 16) using Lipofectamine 2000 (Invitrogen, Carlsbad, CA, USA) according to the manufacturer’s protocol. To select stably integrated cells, the transfected cells were cultured with G418 500 µg/ml (Neomycin, Sigma) for 2 weeks. The surviving cells were cultured with a 1/10 concentration of G418 for several days until colony‐forming units were observed. The surviving colonies were then collected for use in experiments. Melan-A melanocytes that stably expressed luciferase were cultured in serum-free media and treated with or without Dex for 72 h. The supernatants were obtained, and luciferase activity was measured. Relative Gaussia luciferase levels were determined using the BioLux Gaussia Luciferase Assay Kit (New England Biolabs, Beverly, MA, USA) according to the manufacturer’s protocol and were quantitated using a microplate reader (TECAN, Männedorf, Switzerland).

### Chromatin immunoprecipitation (ChIP) assay

Melan-a melanocytes were fixed with 1% formaldehyde at room temperature for 15 min, followed by the addition of glycine (0.125 M) for 5 min to quench. The harvested cells and precipitated pellets were rinsed twice with ice-cold PBS, and were then resuspended in ice-cold cell lysis buffer (1% SDS, 10 mM EDTA, 50 mM Tris–HCl at pH 8.1 with PIC). The samples were then incubated on ice for 15 min, and then were centrifuged at 13,000 rpm for 1 min. The cell pellets were sonicated to < 500 bp size using a Branson Digital Sonifier SFX 550 (EMERSON, St. Louis, MO, USA) and were then centrifuged at 13,000 rpm for 10 min at 4 °C. An aliquot of each supernatant from the whole cell extracts was acquired as a control. The supernatants were incubated with each antibody against polymerase2 (Abcam, Cambridge, UK), GR (Cell Signaling Technology) and IgG (Invitrogen) at 4 °C overnight. After centrifugation at 13,000 rpm at 4 °C for 10 min, the aggregated pellets were removed. Next, protein A/G beads were incubated on a rotating platform at 4 °C for 45 min. Each bead was rinsed 5 times with ice-cold IP buffer to eliminate non-specific binding. The precipitated material was reverse-crosslinked and boiled with Chelex-100 10% (Sigma) for 10 min, after which the tube was briefly centrifuged to acquire the supernatant. The DNA samples obtained were used for PCR and were analyzed by electrophoresis on 2% agarose gels.

### Electrophoretic mobility shift assay (EMSA)

Double-stranded oligonucleotides of the glucocorticoid response element from the ‘GR consensus and mutant Oligonucleotides’ were purchased from Santa Cruz. IR9 and Mutant IR9 were synthesized and purchased from Bioneer. Each sequence was as follows:

GRE consensus sense 5′-[biotin] GACCCTAGAGGATCT**GT**ACAGGAT**GT**TCTAGAT-3ʹ and antisense 5ʹ-[biotin] ATCTAGAACATCCTGTACAGATCCTCTAGGGTC-3ʹ

GRE mutant sense (Cold) 5ʹ- GACCCTAGAGGATCT**CA**ACAGGAT**CA**TCTAGAT-3ʹ and antisense 5ʹ-ATCTAGATGATCCTGTTGAGATCCTCTAGGGTC-3ʹ

IR9 sense 5ʹ-[biotin] GCTTGGGGACAAGGTGCCTCTGTCCTCCA-3ʹ and antisense 5ʹ-[biotin] TGGAGGACAGAGGCACCTTGTCCCCAAGC -3ʹ

Mutant IR9 (M-IR9) sense 5ʹ-[biotin] GCTTGGGG***GT***AAGGT***AT***CTC***CGC***CCTCCA -3ʹ and antisense 5ʹ-[biotin] TGGAGG***GCG***GAG***AT***ACCTT***AC***CCCCAAGC -3ʹ

Each sequence competitor, which was not labelled with biotin, was used at a 50-fold higher concentration than the labelled sequence. EMSA were performed using a LightShift Chemiluminescent EMSA Kit (Thermo) according to the manufacturer’s instructions. Briefly, binding was reacted with 10 μg Dex treated nuclear extract in 10 mM Tris, 50 mM KCl, 1 mM DTT, 10% glycerol, 5 mM MgCl_2_, 0.05% NP-40, and 1 pmol of the oligonucleotide probe. The mixtures were incubated for 1 h at 4 °C. Specific binding was confirmed using a 50-fold excess of unlabeled probe as a specific competitor. Protein-DNA complexes were separated on 6% non-denaturing acrylamide gels at 100 V in TBE buffer (Invitrogen). The gel complexes were transferred to positively charged nylon membranes (Thermo) and were cross-linked using a UVC lamp for 15 min. Gel shifts were visualized with streptavidin-HRP followed by chemiluminescent substrate.

## Supplementary Information

Supplementary Information 1.

Supplementary Information 2.
