# Supplementary material for: Regulation of Melanophilin (Mlph) gene expression by the glucocorticoid receptor (GR)
Source: Sci Rep. 2021 Aug 19;11:16813. doi: 10.1038/s41598-021-96276-w (PMC8376885; doi:10.1038/s41598-021-96276-w)
Supplement: Supplementary file 1 — Supplementary Information 1. [file 41598_2021_96276_MOESM1_ESM.pptx]

## Slide 1
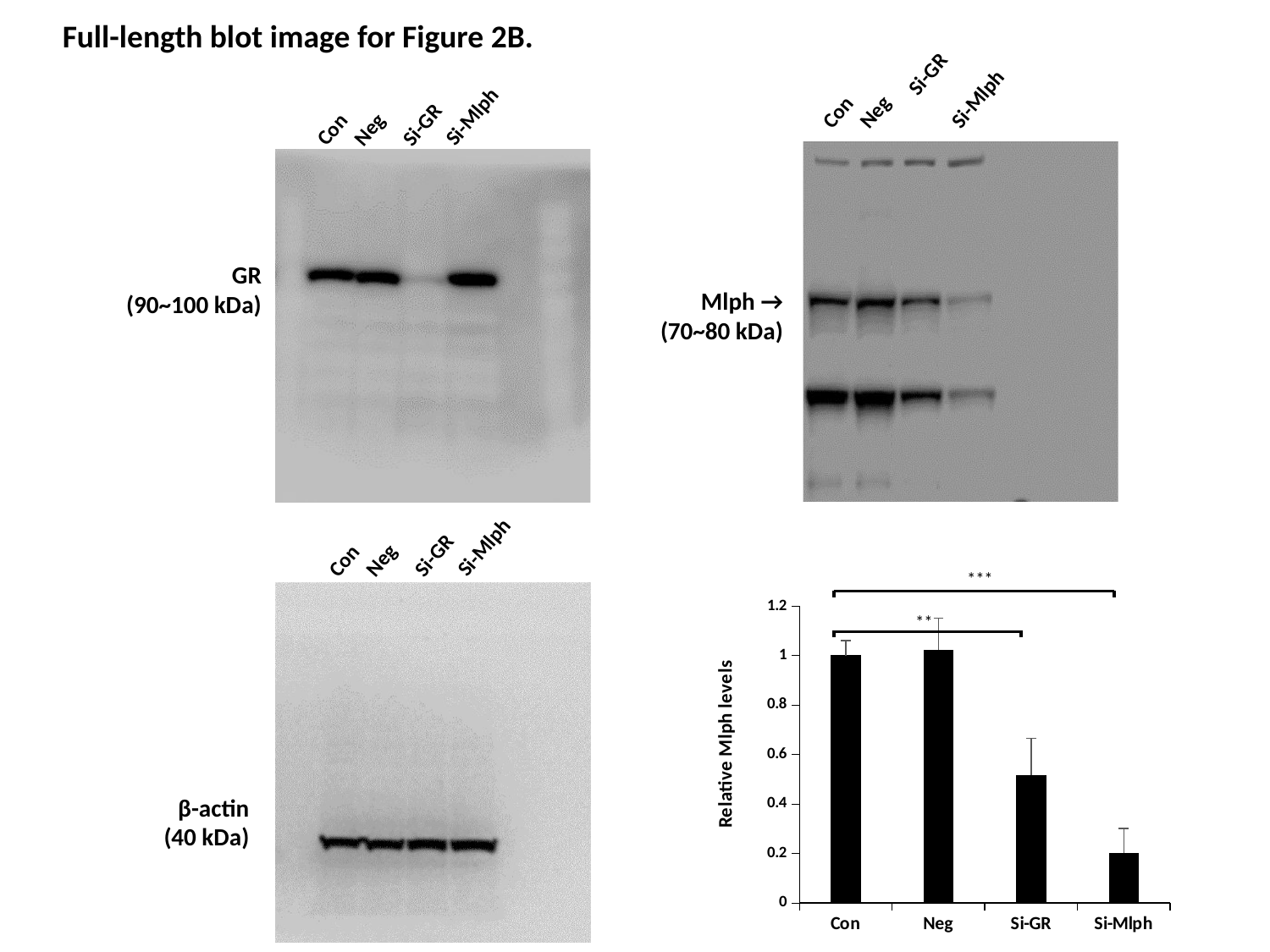

Full-length blot image for Figure 2B.
Si-GR
Si-Mlph
Neg
Con
Si-Mlph
Si-GR
Neg
Con
GR
(90~100 kDa)
Mlph →
(70~80 kDa)
Si-Mlph
Si-GR
Neg
Con
***
### Chart
| Category | |
|---|---|
| Con | 1.0 |
| Neg | 1.021994799394251 |
| Si-GR | 0.5161238206353409 |
| Si-Mlph | 0.20169474714278554 |**
β-actin
(40 kDa)

## Slide 2
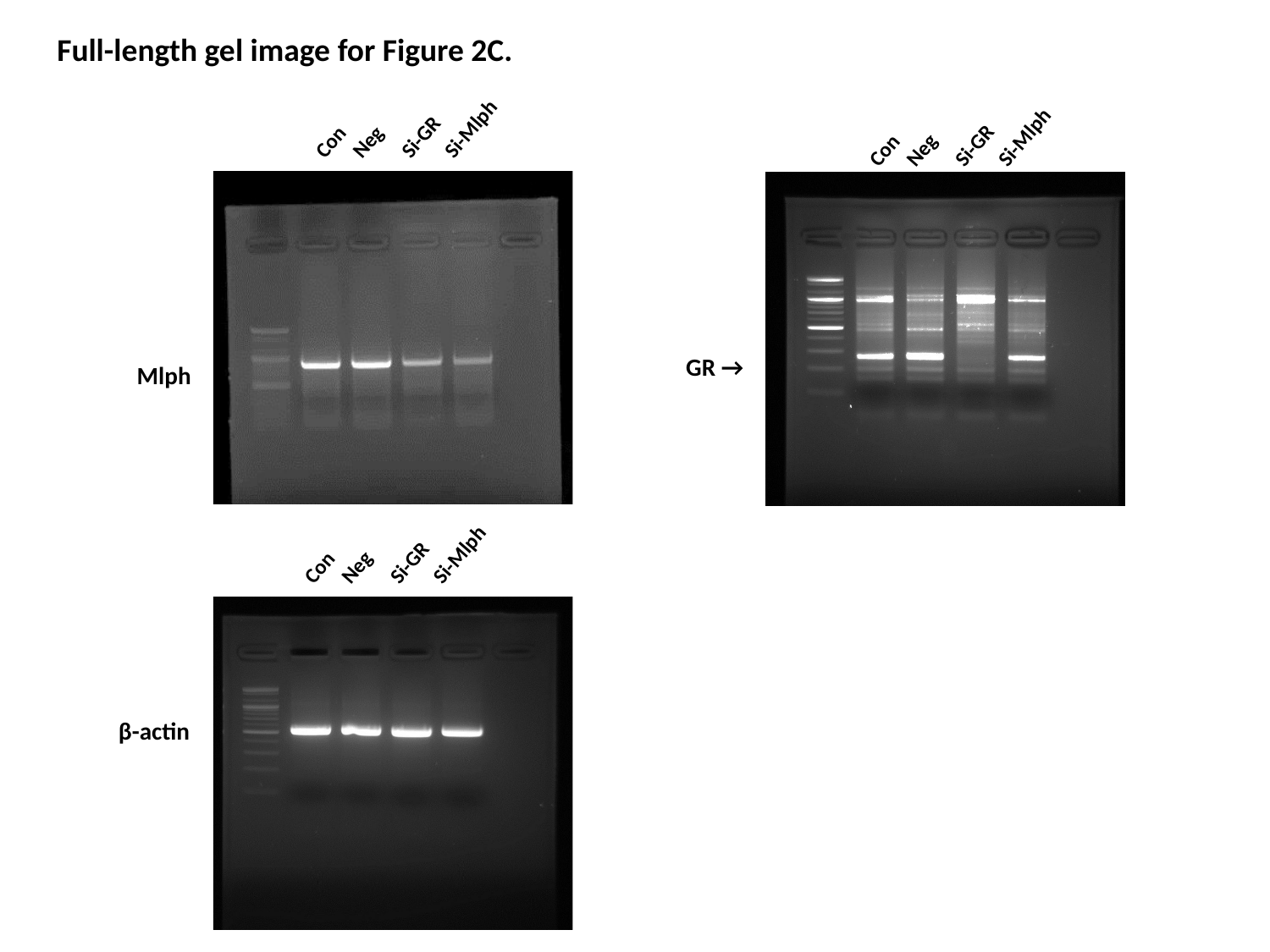

Full-length gel image for Figure 2C.
Si-Mlph
Si-GR
Si-Mlph
Neg
Con
Si-GR
Neg
Con
GR →
Mlph
Si-Mlph
Si-GR
Neg
Con
β-actin

## Slide 3
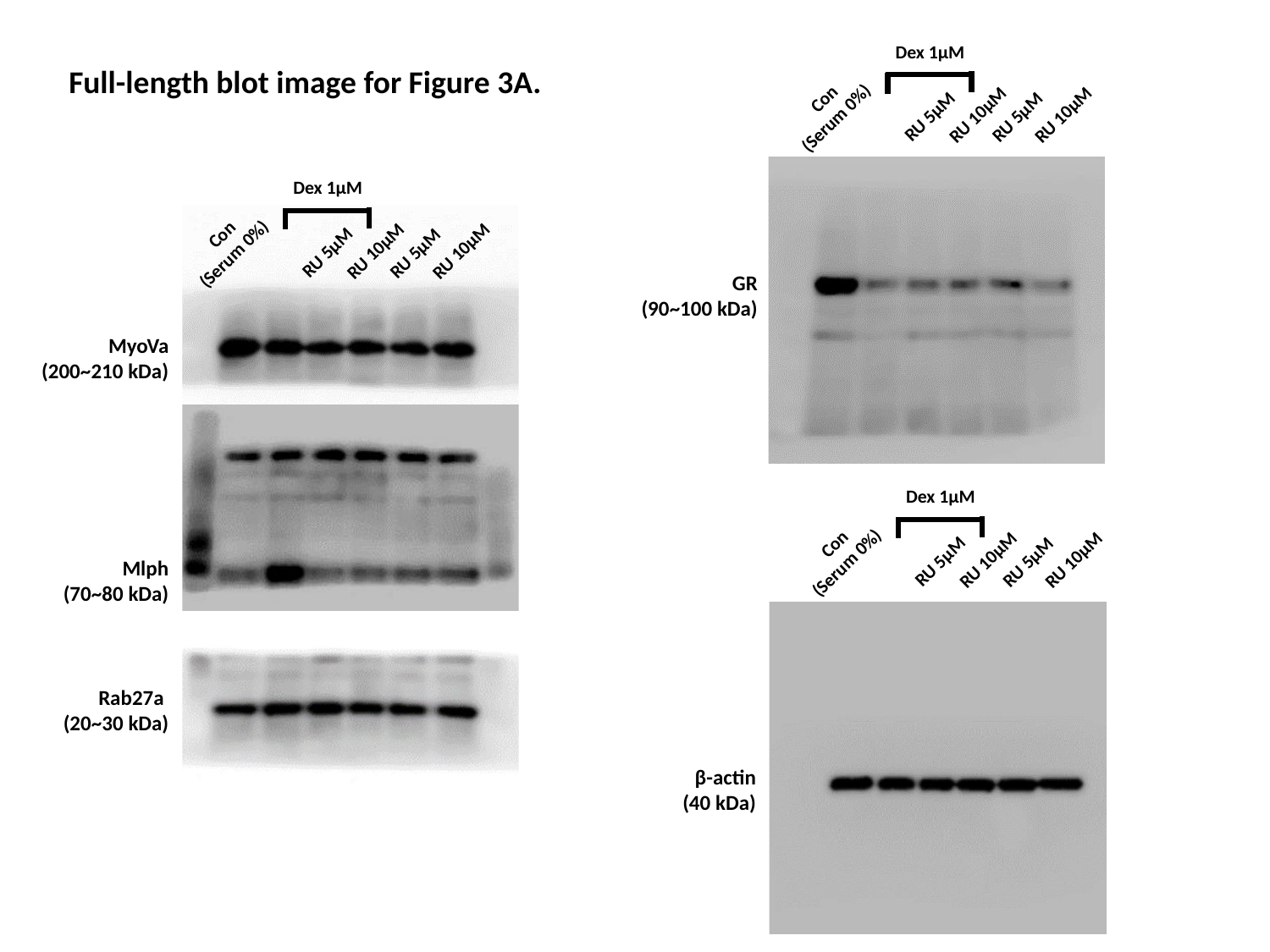

Dex 1µM
Full-length blot image for Figure 3A.
 Con
(Serum 0%)
RU 10µM
RU 10µM
RU 5µM
RU 5µM
Dex 1µM
 Con
(Serum 0%)
RU 10µM
RU 10µM
RU 5µM
RU 5µM
GR
(90~100 kDa)
MyoVa
(200~210 kDa)
Dex 1µM
 Con
(Serum 0%)
RU 10µM
RU 10µM
RU 5µM
RU 5µM
Mlph
(70~80 kDa)
Rab27a
(20~30 kDa)
β-actin
(40 kDa)

## Slide 4
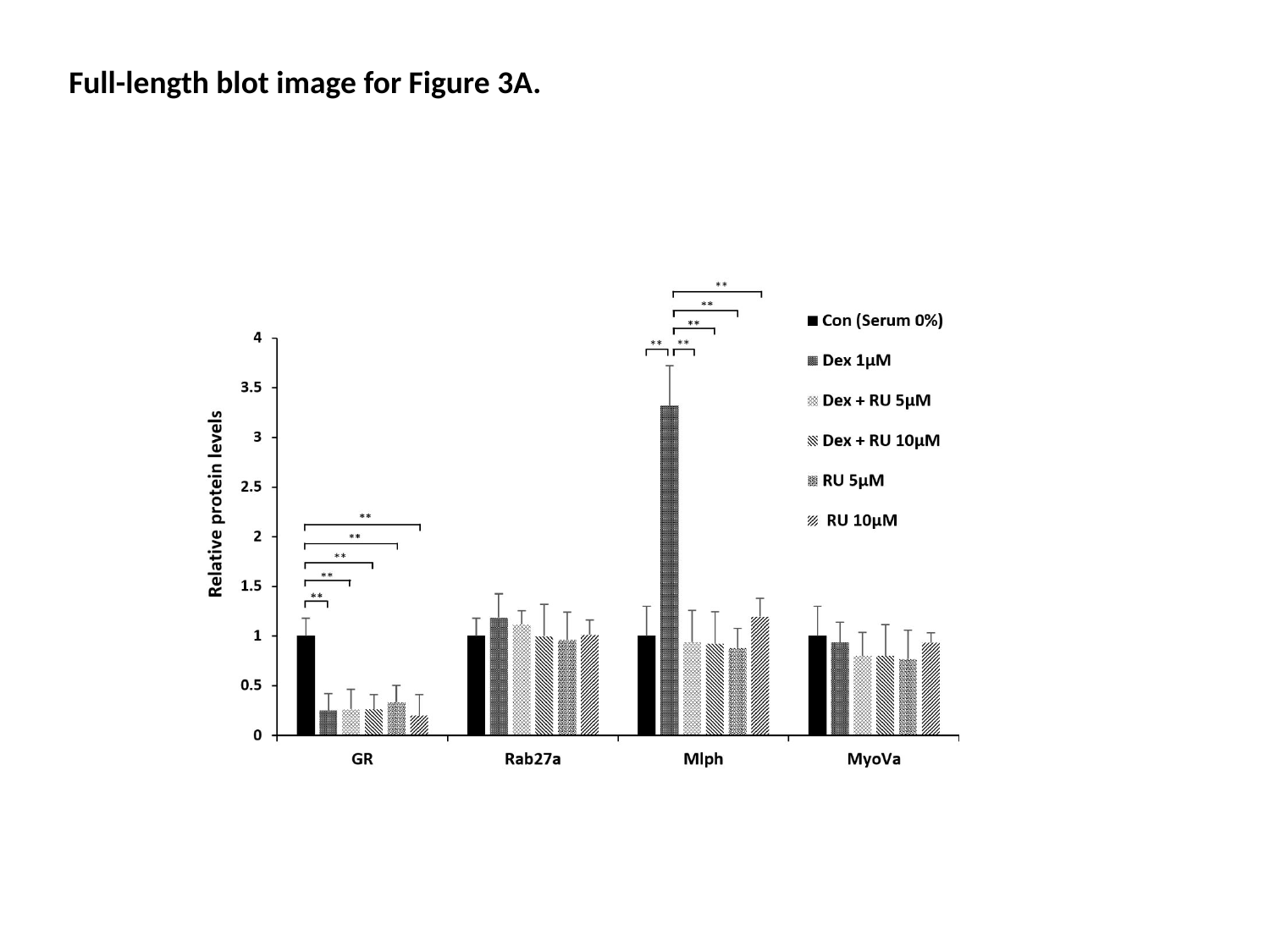

Full-length blot image for Figure 3A.
**
**
### Chart
| Category | Con (Serum 0%) | Dex 1μM | Dex + RU 5μM | Dex + RU 10μM | RU 5μM | RU 10μM |
|---|---|---|---|---|---|---|
| GR | 1.0 | 0.25093973999577374 | 0.2627198828236767 | 0.2611929235238815 | 0.33206282021983047 | 0.20286703216513186 |
| Rab27a | 1.0 | 1.1840947221974607 | 1.1137339306862608 | 0.9989576435493525 | 0.9601095005539496 | 1.0113512709004546 |
| Mlph | 1.0 | 3.3191258890745297 | 0.9378219994535268 | 0.9242520595441086 | 0.8788376752761243 | 1.197185076214976 |
| MyoVa | 1.0 | 0.9361466477147259 | 0.7960153858237898 | 0.7972931912668197 | 0.7672177358562191 | 0.9310616980731148 |**
**
**
**
**
**
**
**

## Slide 5
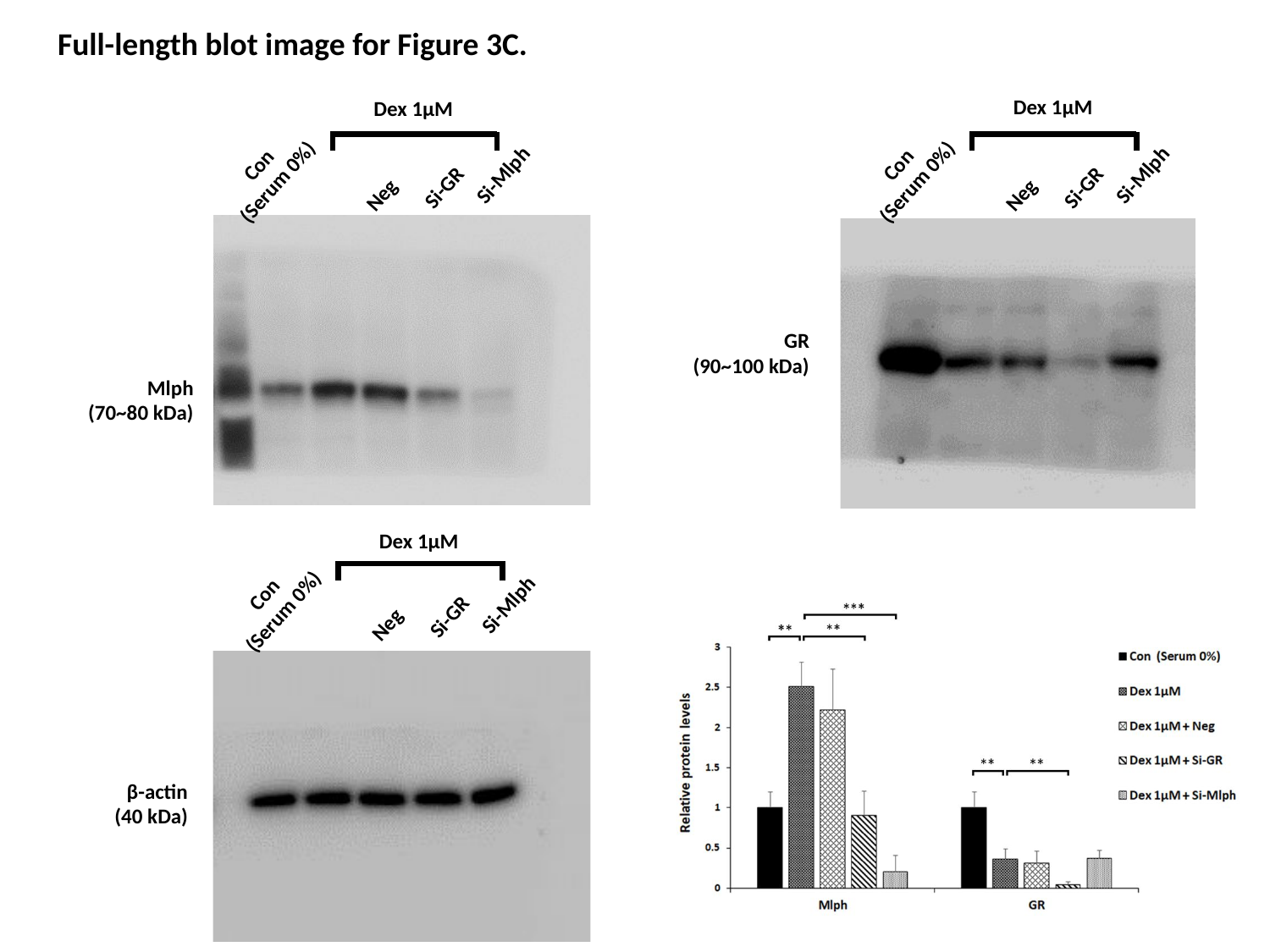

Full-length blot image for Figure 3C.
Dex 1µM
Dex 1µM
Con
(Serum 0%)
Con
(Serum 0%)
Si-Mlph
Si-Mlph
Si-GR
Si-GR
Neg
Neg
GR
(90~100 kDa)
Mlph
(70~80 kDa)
Dex 1µM
Con
(Serum 0%)
Si-Mlph
Si-GR
Neg
β-actin
(40 kDa)

## Slide 6
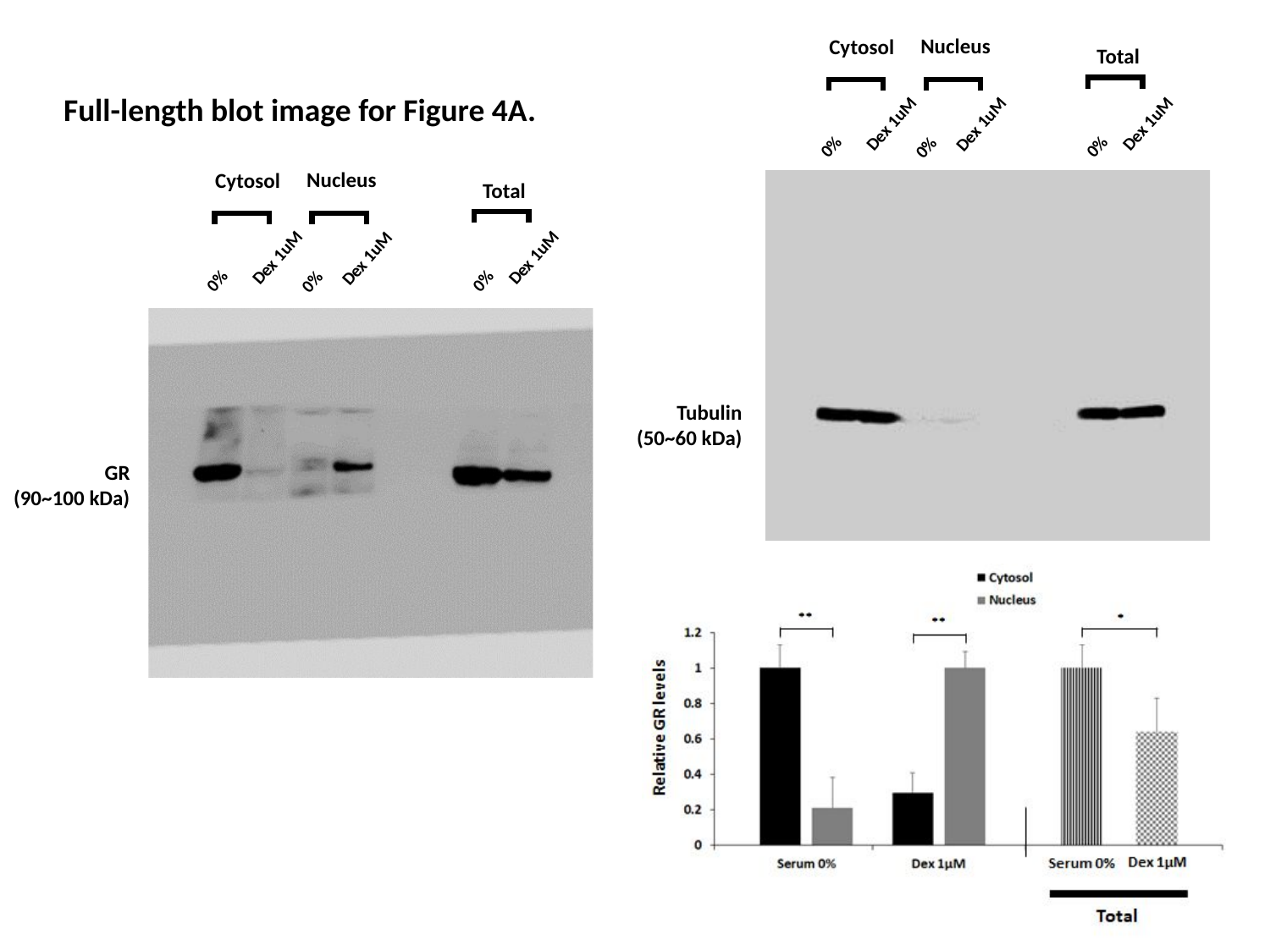

Nucleus
Cytosol
Total
Full-length blot image for Figure 4A.
Dex 1uM
Dex 1uM
Dex 1uM
0%
0%
0%
Nucleus
Cytosol
Total
Dex 1uM
Dex 1uM
Dex 1uM
0%
0%
0%
Tubulin
(50~60 kDa)
GR
(90~100 kDa)

## Slide 7
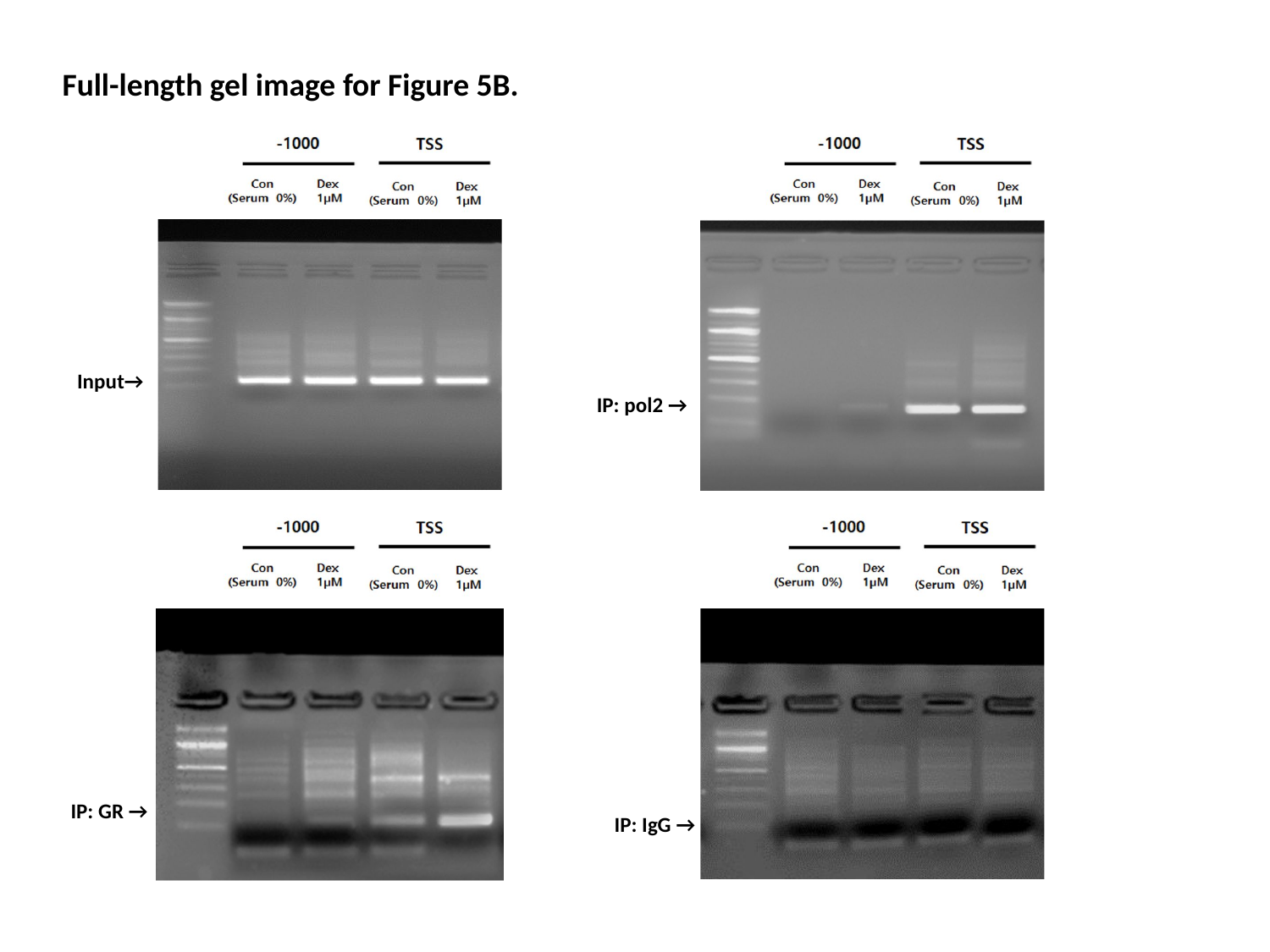

Full-length gel image for Figure 5B.
Input→
IP: pol2 →
IP: GR →
IP: IgG →

## Slide 8
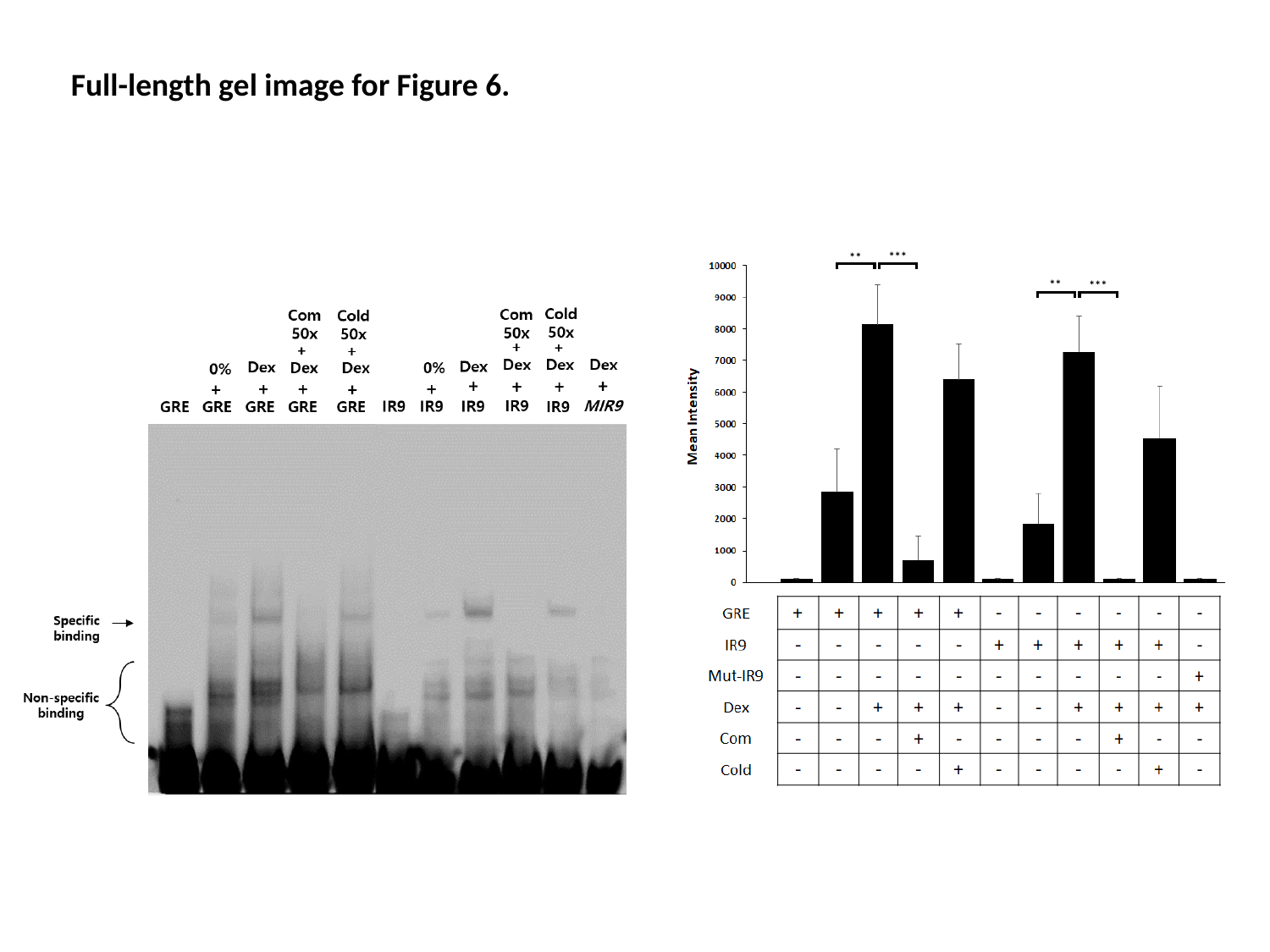

Full-length gel image for Figure 6.
***
**
**
***

## Slide 9
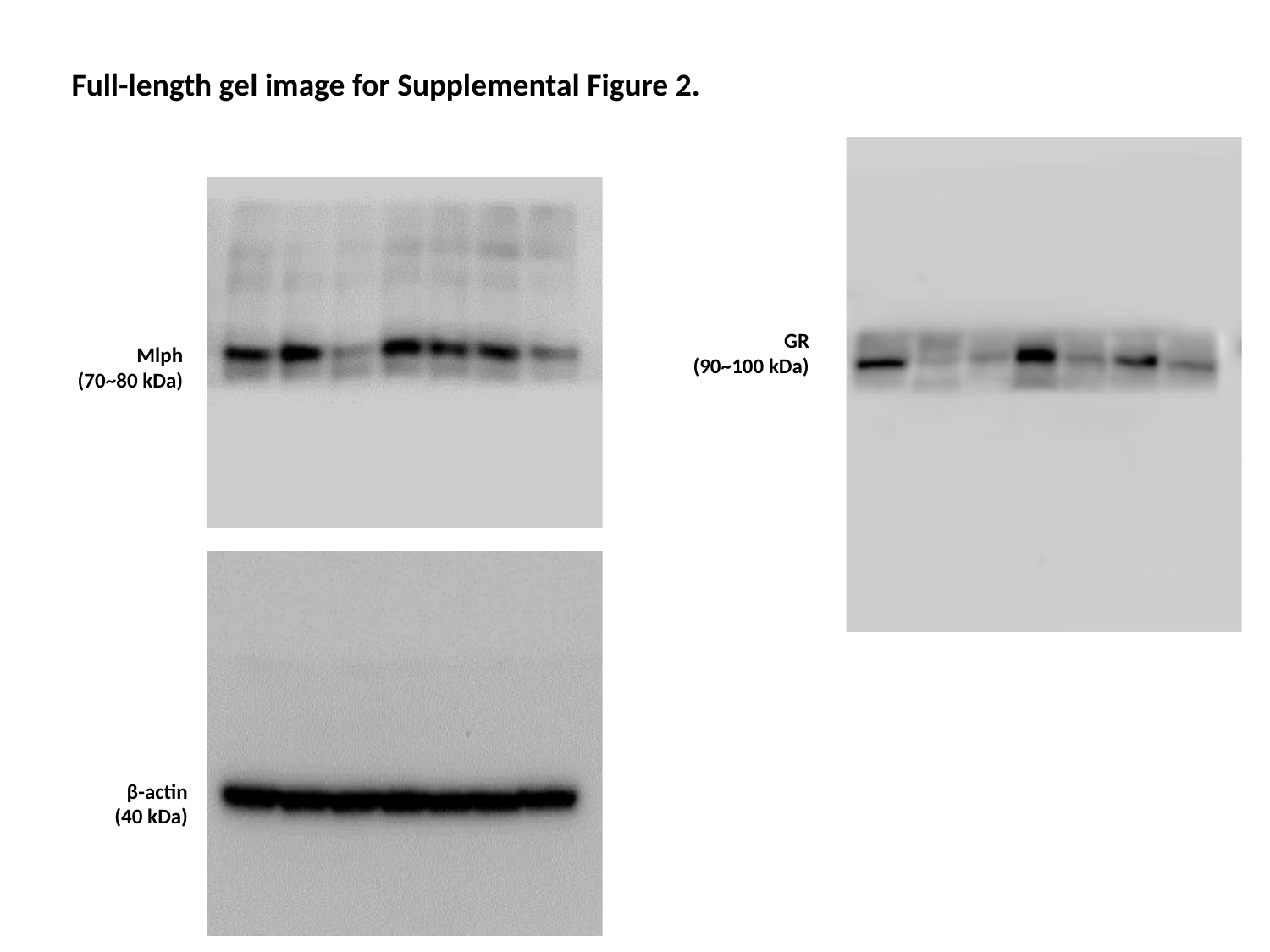

Full-length gel image for Supplemental Figure 2.
GR
(90~100 kDa)
Mlph
(70~80 kDa)
β-actin
(40 kDa)
