# Supplementary material for: Regulation of Melanophilin (Mlph) gene expression by the glucocorticoid receptor (GR)
Source: Sci Rep. 2021 Aug 19;11:16813. doi: 10.1038/s41598-021-96276-w (PMC8376885; doi:10.1038/s41598-021-96276-w)
Supplement: Supplementary file 2 — Supplementary Information 2. [file 41598_2021_96276_MOESM2_ESM.pptx]

## Slide 1
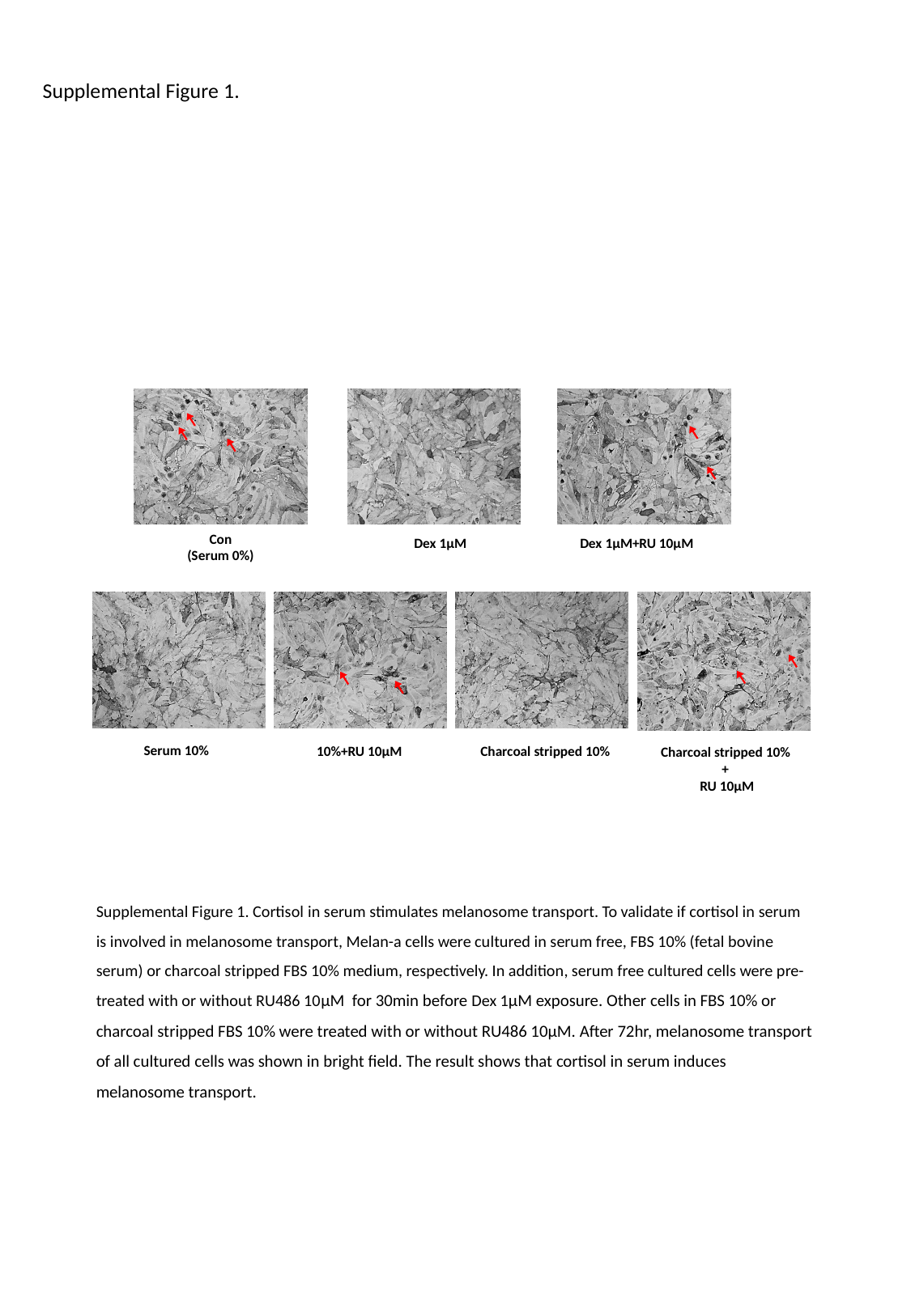

Supplemental Figure 1.
Con
(Serum 0%)
Dex 1µM
Dex 1µM+RU 10µM
Serum 10%
10%+RU 10µM
Charcoal stripped 10%
Charcoal stripped 10%
+
RU 10µM
Supplemental Figure 1. Cortisol in serum stimulates melanosome transport. To validate if cortisol in serum is involved in melanosome transport, Melan-a cells were cultured in serum free, FBS 10% (fetal bovine serum) or charcoal stripped FBS 10% medium, respectively. In addition, serum free cultured cells were pre-treated with or without RU486 10µM for 30min before Dex 1µM exposure. Other cells in FBS 10% or charcoal stripped FBS 10% were treated with or without RU486 10µM. After 72hr, melanosome transport of all cultured cells was shown in bright field. The result shows that cortisol in serum induces melanosome transport.

## Slide 2
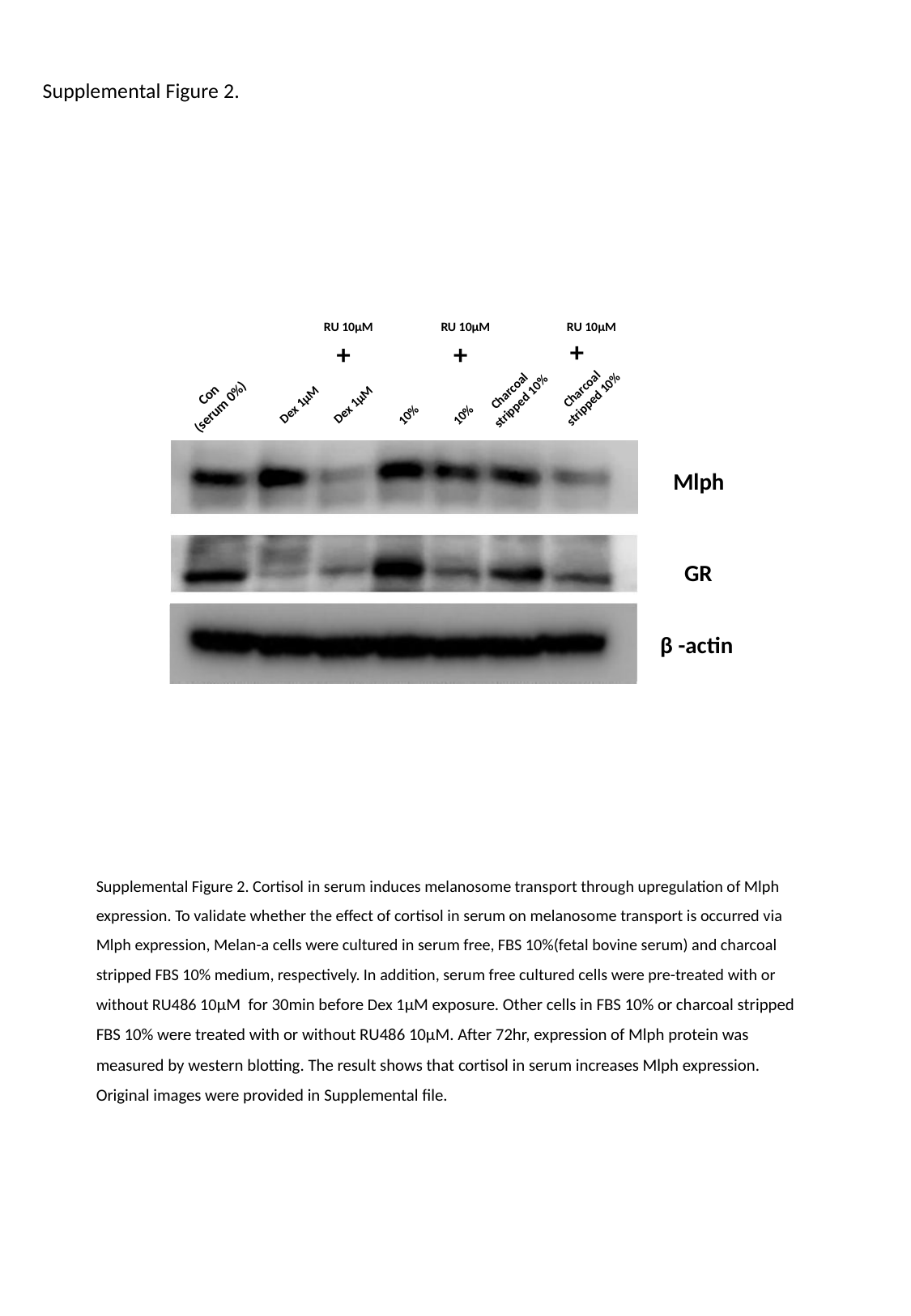

Supplemental Figure 2.
RU 10µM
RU 10µM
RU 10µM
+
+
+
Charcoal
stripped 10%
Charcoal
stripped 10%
Con
(serum 0%)
Dex 1µM
Dex 1µM
10%
10%
Mlph
GR
β -actin
Supplemental Figure 2. Cortisol in serum induces melanosome transport through upregulation of Mlph expression. To validate whether the effect of cortisol in serum on melanosome transport is occurred via Mlph expression, Melan-a cells were cultured in serum free, FBS 10%(fetal bovine serum) and charcoal stripped FBS 10% medium, respectively. In addition, serum free cultured cells were pre-treated with or without RU486 10µM for 30min before Dex 1µM exposure. Other cells in FBS 10% or charcoal stripped FBS 10% were treated with or without RU486 10µM. After 72hr, expression of Mlph protein was measured by western blotting. The result shows that cortisol in serum increases Mlph expression. Original images were provided in Supplemental file.

## Slide 3
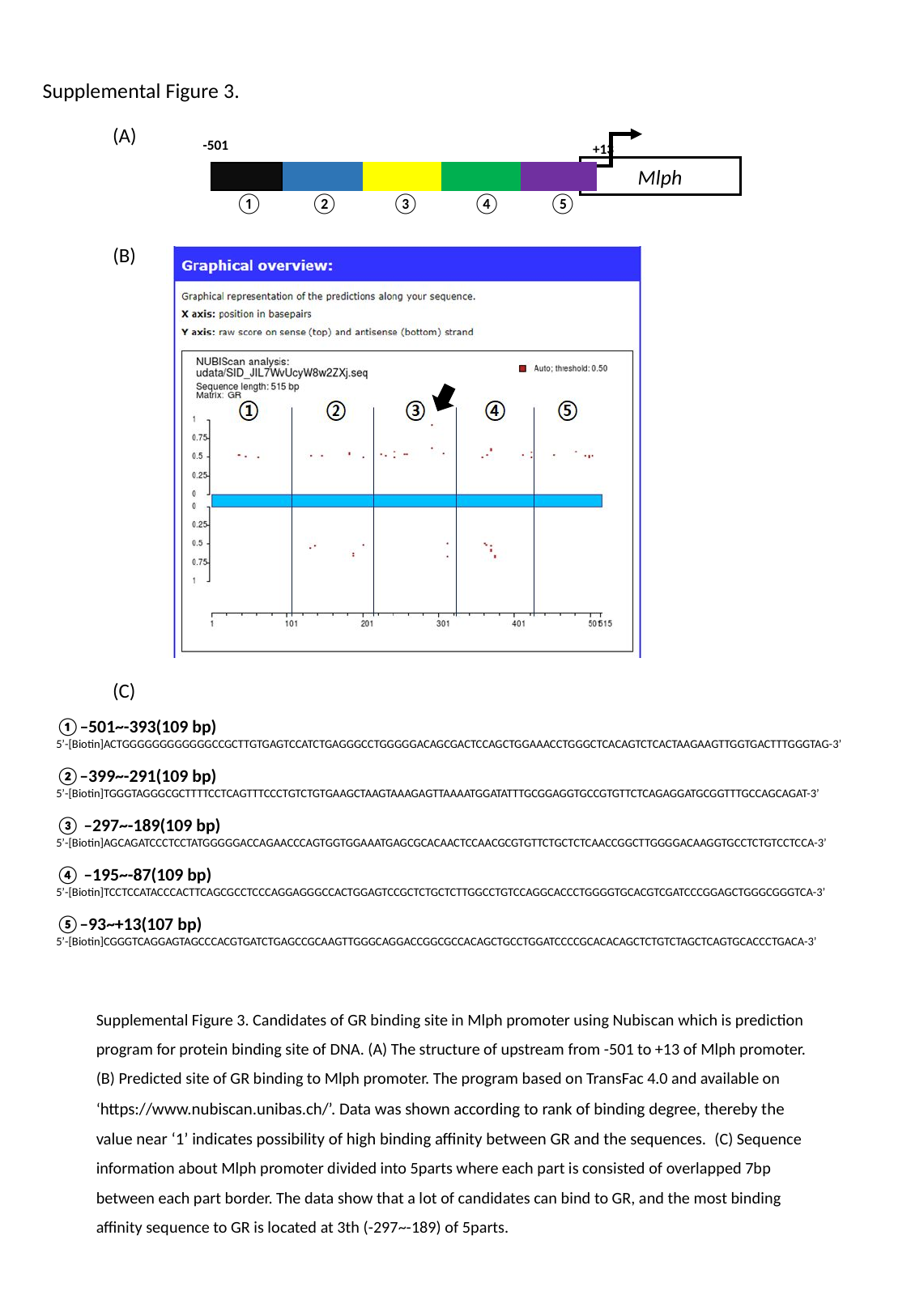

Supplemental Figure 3.
(A)
-501
+13
Mlph
①
②
⑤
③
④
(B)
(C)
①–501~-393(109 bp)
5’-[Biotin]ACTGGGGGGGGGGGGCCGCTTGTGAGTCCATCTGAGGGCCTGGGGGACAGCGACTCCAGCTGGAAACCTGGGCTCACAGTCTCACTAAGAAGTTGGTGACTTTGGGTAG-3’
②–399~-291(109 bp)
5’-[Biotin]TGGGTAGGGCGCTTTTCCTCAGTTTCCCTGTCTGTGAAGCTAAGTAAAGAGTTAAAATGGATATTTGCGGAGGTGCCGTGTTCTCAGAGGATGCGGTTTGCCAGCAGAT-3’
③ –297~-189(109 bp)
5’-[Biotin]AGCAGATCCCTCCTATGGGGGACCAGAACCCAGTGGTGGAAATGAGCGCACAACTCCAACGCGTGTTCTGCTCTCAACCGGCTTGGGGACAAGGTGCCTCTGTCCTCCA-3’
④ –195~-87(109 bp)
5’-[Biotin]TCCTCCATACCCACTTCAGCGCCTCCCAGGAGGGCCACTGGAGTCCGCTCTGCTCTTGGCCTGTCCAGGCACCCTGGGGTGCACGTCGATCCCGGAGCTGGGCGGGTCA-3’
⑤–93~+13(107 bp)
5’-[Biotin]CGGGTCAGGAGTAGCCCACGTGATCTGAGCCGCAAGTTGGGCAGGACCGGCGCCACAGCTGCCTGGATCCCCGCACACAGCTCTGTCTAGCTCAGTGCACCCTGACA-3’
Supplemental Figure 3. Candidates of GR binding site in Mlph promoter using Nubiscan which is prediction program for protein binding site of DNA. (A) The structure of upstream from -501 to +13 of Mlph promoter. (B) Predicted site of GR binding to Mlph promoter. The program based on TransFac 4.0 and available on ‘https://www.nubiscan.unibas.ch/’. Data was shown according to rank of binding degree, thereby the value near ‘1’ indicates possibility of high binding affinity between GR and the sequences. (C) Sequence information about Mlph promoter divided into 5parts where each part is consisted of overlapped 7bp between each part border. The data show that a lot of candidates can bind to GR, and the most binding affinity sequence to GR is located at 3th (-297~-189) of 5parts.

## Slide 4
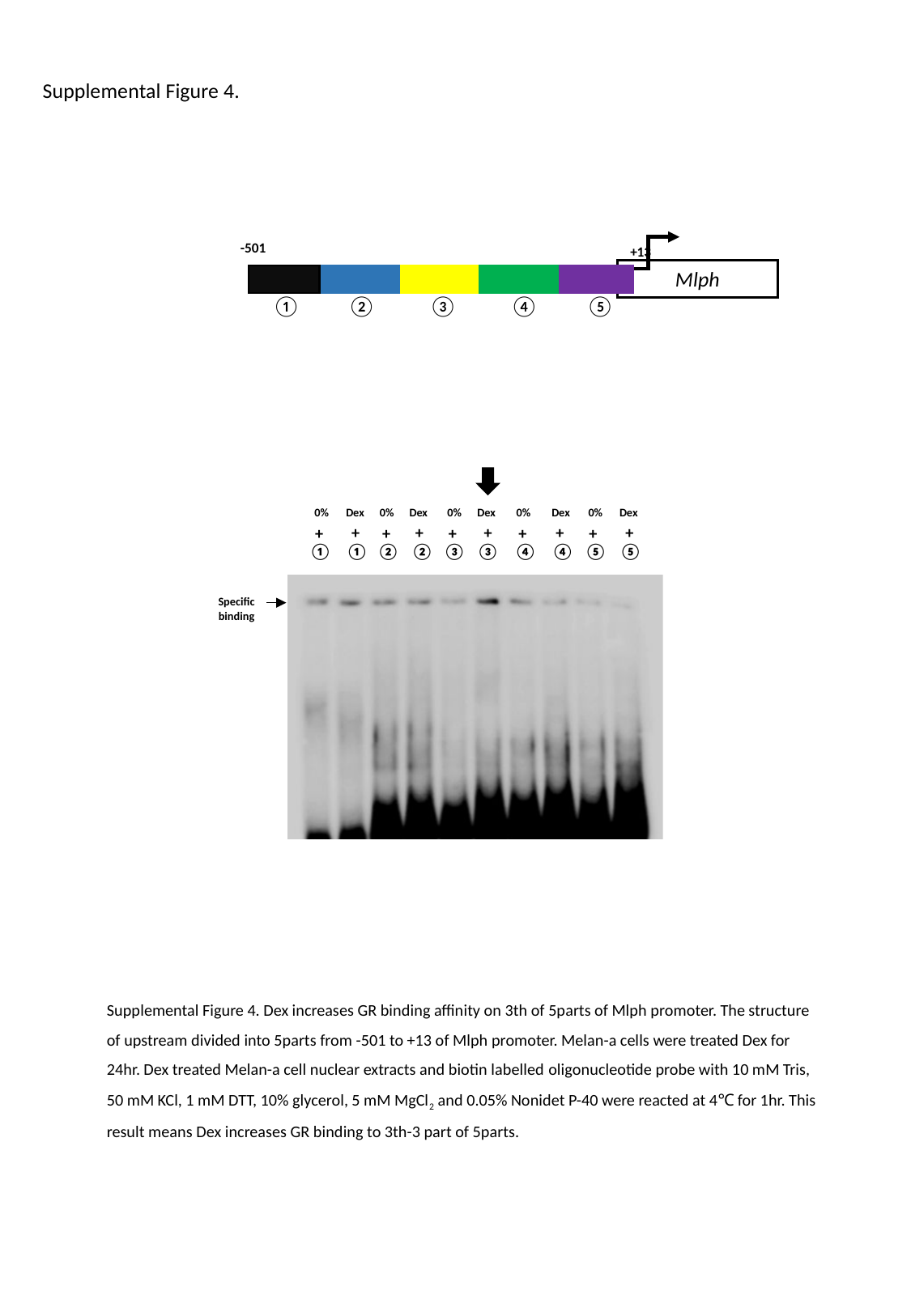

Supplemental Figure 4.
-501
+13
Mlph
①
②
⑤
③
④
 Dex
0%
 Dex
 Dex
 Dex
 Dex
0%
0%
0%
0%
+
+
+
+
+
+
+
+
+
+
①
①
②
②
③
③
④
④
⑤
⑤
Specific
binding
Supplemental Figure 4. Dex increases GR binding affinity on 3th of 5parts of Mlph promoter. The structure of upstream divided into 5parts from -501 to +13 of Mlph promoter. Melan-a cells were treated Dex for 24hr. Dex treated Melan-a cell nuclear extracts and biotin labelled oligonucleotide probe with 10 mM Tris, 50 mM KCl, 1 mM DTT, 10% glycerol, 5 mM MgCl2 and 0.05% Nonidet P-40 were reacted at 4℃ for 1hr. This result means Dex increases GR binding to 3th-3 part of 5parts.

## Slide 5
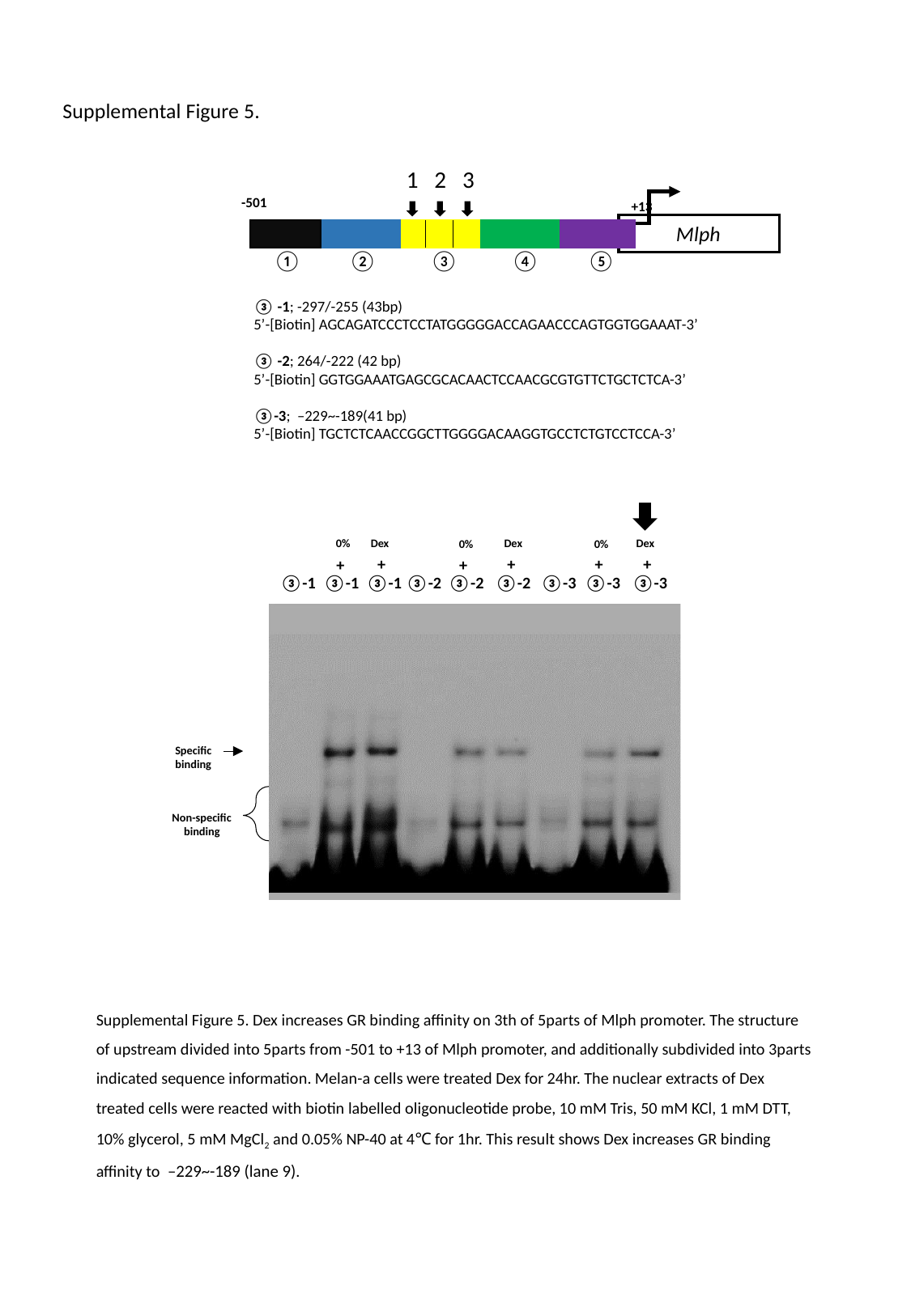

Supplemental Figure 5.
1
2
3
-501
+13
Mlph
①
②
⑤
③
④
③ -1; -297/-255 (43bp)
5’-[Biotin] AGCAGATCCCTCCTATGGGGGACCAGAACCCAGTGGTGGAAAT-3’
③ -2; 264/-222 (42 bp)
5’-[Biotin] GGTGGAAATGAGCGCACAACTCCAACGCGTGTTCTGCTCTCA-3’
③-3; –229~-189(41 bp)
5’-[Biotin] TGCTCTCAACCGGCTTGGGGACAAGGTGCCTCTGTCCTCCA-3’
0%
 Dex
 Dex
 Dex
0%
0%
+
+
+
+
+
+
③-1
③-1
③-1
③-2
③-2
③-2
③-3
③-3
③-3
Specific
binding
Non-specific
binding
Supplemental Figure 5. Dex increases GR binding affinity on 3th of 5parts of Mlph promoter. The structure of upstream divided into 5parts from -501 to +13 of Mlph promoter, and additionally subdivided into 3parts indicated sequence information. Melan-a cells were treated Dex for 24hr. The nuclear extracts of Dex treated cells were reacted with biotin labelled oligonucleotide probe, 10 mM Tris, 50 mM KCl, 1 mM DTT, 10% glycerol, 5 mM MgCl2 and 0.05% NP-40 at 4℃ for 1hr. This result shows Dex increases GR binding affinity to –229~-189 (lane 9).

## Slide 6
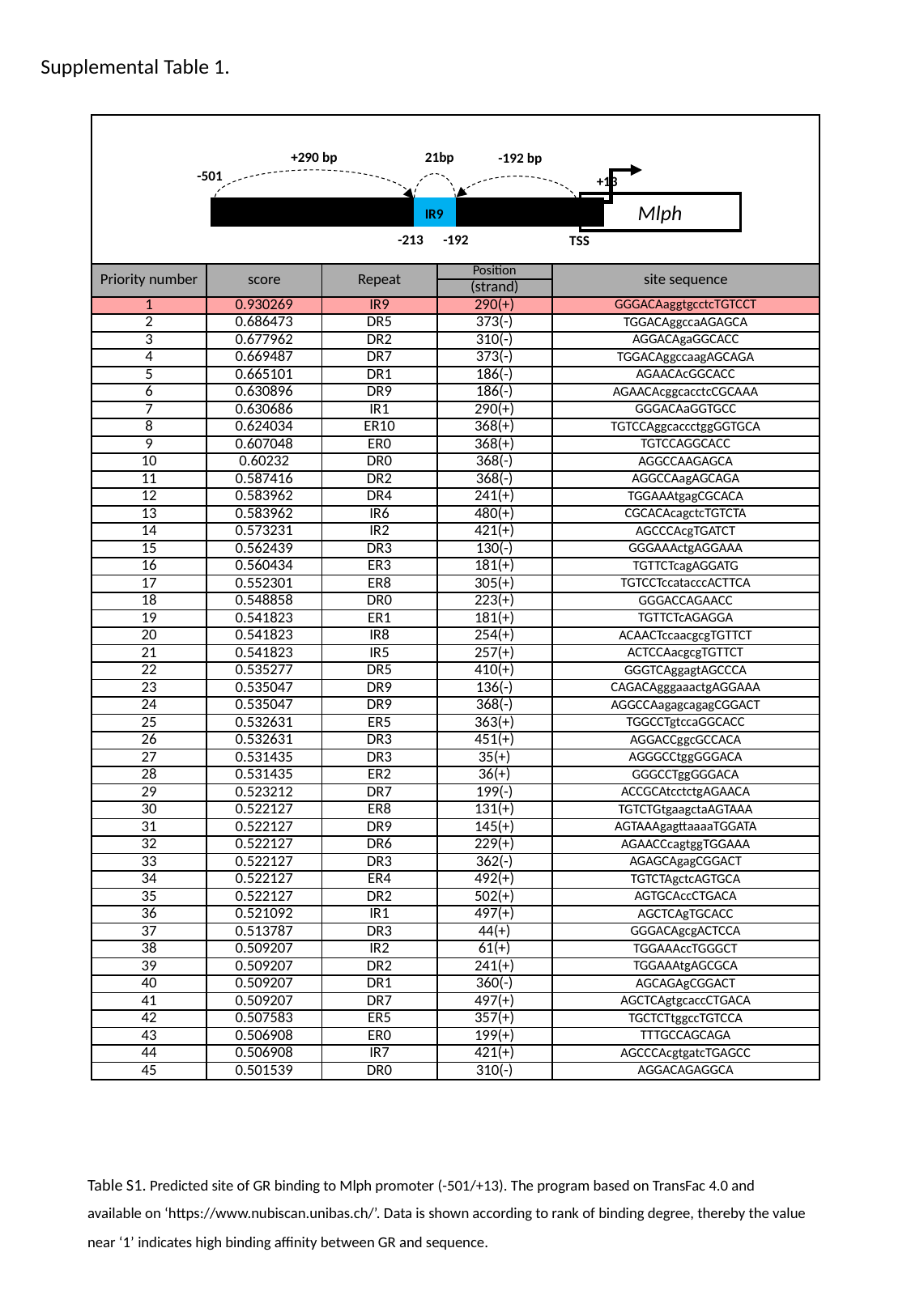

Supplemental Table 1.
| | | | | |
| --- | --- | --- | --- | --- |
| Priority number | score | Repeat | Position | site sequence |
| | | | (strand) | |
| 1 | 0.930269 | IR9 | 290(+) | GGGACAaggtgcctcTGTCCT |
| 2 | 0.686473 | DR5 | 373(-) | TGGACAggccaAGAGCA |
| 3 | 0.677962 | DR2 | 310(-) | AGGACAgaGGCACC |
| 4 | 0.669487 | DR7 | 373(-) | TGGACAggccaagAGCAGA |
| 5 | 0.665101 | DR1 | 186(-) | AGAACAcGGCACC |
| 6 | 0.630896 | DR9 | 186(-) | AGAACAcggcacctcCGCAAA |
| 7 | 0.630686 | IR1 | 290(+) | GGGACAaGGTGCC |
| 8 | 0.624034 | ER10 | 368(+) | TGTCCAggcaccctggGGTGCA |
| 9 | 0.607048 | ER0 | 368(+) | TGTCCAGGCACC |
| 10 | 0.60232 | DR0 | 368(-) | AGGCCAAGAGCA |
| 11 | 0.587416 | DR2 | 368(-) | AGGCCAagAGCAGA |
| 12 | 0.583962 | DR4 | 241(+) | TGGAAAtgagCGCACA |
| 13 | 0.583962 | IR6 | 480(+) | CGCACAcagctcTGTCTA |
| 14 | 0.573231 | IR2 | 421(+) | AGCCCAcgTGATCT |
| 15 | 0.562439 | DR3 | 130(-) | GGGAAActgAGGAAA |
| 16 | 0.560434 | ER3 | 181(+) | TGTTCTcagAGGATG |
| 17 | 0.552301 | ER8 | 305(+) | TGTCCTccatacccACTTCA |
| 18 | 0.548858 | DR0 | 223(+) | GGGACCAGAACC |
| 19 | 0.541823 | ER1 | 181(+) | TGTTCTcAGAGGA |
| 20 | 0.541823 | IR8 | 254(+) | ACAACTccaacgcgTGTTCT |
| 21 | 0.541823 | IR5 | 257(+) | ACTCCAacgcgTGTTCT |
| 22 | 0.535277 | DR5 | 410(+) | GGGTCAggagtAGCCCA |
| 23 | 0.535047 | DR9 | 136(-) | CAGACAgggaaactgAGGAAA |
| 24 | 0.535047 | DR9 | 368(-) | AGGCCAagagcagagCGGACT |
| 25 | 0.532631 | ER5 | 363(+) | TGGCCTgtccaGGCACC |
| 26 | 0.532631 | DR3 | 451(+) | AGGACCggcGCCACA |
| 27 | 0.531435 | DR3 | 35(+) | AGGGCCtggGGGACA |
| 28 | 0.531435 | ER2 | 36(+) | GGGCCTggGGGACA |
| 29 | 0.523212 | DR7 | 199(-) | ACCGCAtcctctgAGAACA |
| 30 | 0.522127 | ER8 | 131(+) | TGTCTGtgaagctaAGTAAA |
| 31 | 0.522127 | DR9 | 145(+) | AGTAAAgagttaaaaTGGATA |
| 32 | 0.522127 | DR6 | 229(+) | AGAACCcagtggTGGAAA |
| 33 | 0.522127 | DR3 | 362(-) | AGAGCAgagCGGACT |
| 34 | 0.522127 | ER4 | 492(+) | TGTCTAgctcAGTGCA |
| 35 | 0.522127 | DR2 | 502(+) | AGTGCAccCTGACA |
| 36 | 0.521092 | IR1 | 497(+) | AGCTCAgTGCACC |
| 37 | 0.513787 | DR3 | 44(+) | GGGACAgcgACTCCA |
| 38 | 0.509207 | IR2 | 61(+) | TGGAAAccTGGGCT |
| 39 | 0.509207 | DR2 | 241(+) | TGGAAAtgAGCGCA |
| 40 | 0.509207 | DR1 | 360(-) | AGCAGAgCGGACT |
| 41 | 0.509207 | DR7 | 497(+) | AGCTCAgtgcaccCTGACA |
| 42 | 0.507583 | ER5 | 357(+) | TGCTCTtggccTGTCCA |
| 43 | 0.506908 | ER0 | 199(+) | TTTGCCAGCAGA |
| 44 | 0.506908 | IR7 | 421(+) | AGCCCAcgtgatcTGAGCC |
| 45 | 0.501539 | DR0 | 310(-) | AGGACAGAGGCA |
+290 bp
21bp
-192 bp
-501
+13
Mlph
IR9
-213
-192
TSS
Table S1. Predicted site of GR binding to Mlph promoter (-501/+13). The program based on TransFac 4.0 and available on ‘https://www.nubiscan.unibas.ch/’. Data is shown according to rank of binding degree, thereby the value near ‘1’ indicates high binding affinity between GR and sequence.
